# Identity-based controlled delegated outsourcing data integrity auditing scheme

**DOI:** 10.1038/s41598-024-58325-y

**Published:** 2024-03-30

**Authors:** Jianming Du, Guofang Dong, Juangui Ning, Zhengnan Xu, Ruicheng Yang

**Affiliations:** 1 School of Electrical and Information Technology, Yunnan Minzu University, Kunming, 650504 China; 2 Yunnan Key Laboratory of Unmanned Autonomous System, Yunnan Minzu University, Kunming, 650504 China

**Keywords:** Cloud storage, Integrity auditing, Identity-based encryption, Controlled delegation, Dynamic update, Computer science, Software

## Abstract

With the continuous development of cloud computing, the application of cloud storage has become more and more popular. To ensure the integrity and availability of cloud data, scholars have proposed several cloud data auditing schemes. Still, most need help with outsourced data integrity, controlled outsourcing, and source file auditing. Therefore, we propose a controlled delegation outsourcing data integrity auditing scheme based on the identity-based encryption model. Our proposed scheme allows users to specify a dedicated agent to assist in uploading data to the cloud. These authorized proxies use recognizable identities for authentication and authorization, thus avoiding the need for cumbersome certificate management in a secure distributed computing system. While solving the above problems, our scheme adopts a bucket-based red–black tree structure to efficiently realize the dynamic updating of data, which can complete the updating of data and rebalancing of structural updates constantly and realize the high efficiency of data operations. We define the security model of the scheme in detail and prove the scheme's security under the difficult problem assumption. In the performance analysis section, the proposed scheme is analyzed experimentally in comparison with other schemes, and the results show that the proposed scheme is efficient and secure.

## Introduction

Cloud computing is becoming more widely used in everyday life. It has robust scalability and application requirements and can provide users with new computing resources and data center experience. Cloud storage has many advantages; for example, the utilization of this technology has the potential to alleviate the challenges associated with data administration and upkeep, hence providing users with the convenience of accessing data at any given time and location^1^. Cloud storage has attracted many individuals and organizations with its flexibility, efficiency, and low cost. Cloud storage technology distinguishes itself from traditional storage methods by offering consumers a substantial storage capacity and the convenience of accessing data from various geographical places^2^.

While cloud storage offers many advantages to individuals, it also encounters several challenges along its evolution. One of the most crucial concerns is preserving data integrity and privacy inside cloud computing environments. Since in the cloud storage model, the cloud service provider (CSP) will hold a large amount of users' data centrally, it is very profitable for attackers to attack the CSP, so the CSP can easily become the target of attackers^3^. After uploading data to the cloud, users lose physical control of the data^4^. Despite the proliferation of various cloud data auditing proposals^5–8^, instances of cloud data leakage and tampering continue to occur sporadically, e.g., cloud service providers will maintain their reputation by hiding data incidents^9^.

At the same time, we note that some existing cloud data auditing solutions lack a controlled outsourced delegation approach. In real scenarios, many large organizations providing cloud services (e.g., Google, Apple, Amazon, etc.) can use their signature information to appoint an proxy to help them upload or modify data information. The majority of current cloud data integrity auditing systems rely on the use of a public auditing mechanism^10^. In this mechanism, users often delegate audit tasks to third-party organizations in order to alleviate their computational burden. Consequently, it becomes crucial for the data auditing process to prioritize safeguarding data privacy. Nevertheless, many cloud data auditing schemes^5,8,12–16^ rely on traditional public key infrastructure (PKI) technologies that provide intricate challenges in certificate management. In existing schemes, certificate management indeed presents complex challenges. Many cloud data auditing schemes utilize PKI technologies to verify the identity of cloud service providers and ensure data integrity. However, certificate management involves intricate operations such as certificate generation, issuance, distribution, renewal, and revocation. These operations require maintaining a large number of certificates and ensuring their validity and security. On the other hand, the identity-based encryption (IBE) can effectively address the complex certificate management problem. In the current solutions for cloud data auditing, there are several issues that need to be addressed. Outsourced data integrity is a significant concern in cloud computing. When users store their data in the cloud, they need assurance that the data remains intact and unaltered during its storage and retrieval. Existing solutions often rely on cryptographic techniques, such as hash functions or digital signatures, to verify data integrity. However, these solutions may still be vulnerable to attacks or manipulation by malicious cloud service providers or unauthorized users. Controlled outsourcing is another challenge in cloud storage. Users may want to delegate specific tasks, such as uploading data, to dedicated agents or proxies. However, existing schemes often lack efficient mechanisms for managing and controlling these delegated operations. This can lead to security risks, as unauthorized or malicious agents may gain access to sensitive data or perform unauthorized actions on behalf of the users. Source file auditing is also an important aspect of cloud data auditing. Users need to ensure that the data uploaded to the cloud is consistent with the original source files and that the cloud storage service faithfully reflects any updates or modifications made to the data. Existing solutions may not provide robust mechanisms for auditing the integrity of source files, leading to potential inconsistencies or discrepancies between the local copies and the cloud-stored data. Secondly, the remotely stored data can be accessed and updated by the user side, such as data modification, deletion, insertion, and other operations. In order to ensure that the data is updated promptly and that the user can obtain real-time update information from the cloud server to grasp the dynamics of the monitoring data accurately, it is necessary to realize the efficient updating operation of the data. Therefore, the motivation behind the proposed scheme is to address these issues and provide a controlled delegation outsourcing data integrity auditing solution based on the identity-based encryption model. By allowing users to specify dedicated agents for data upload and employing recognizable identities for authentication and authorization, the proposed scheme aims to overcome the limitations of current solutions and provide a more secure and efficient approach to cloud data auditing.

In this paper, we provide a proposed system for identity-based controlled delegated outsourced data integrity auditing to overcome the difficulties mentioned above. Our contribution is summarized as follows, with due consideration given to the auditing method's efficacy and security.

1. We propose an identity-based controlled delegation outsourcing mechanism. Authorized agents can securely outsource data to dishonest cloud service providers, while unauthorized agents cannot outsource users' data. This identity-based delegation mechanism can be extended to multi-user environments.
2. Our proposed scheme can effectively verify the integrity of outsourced documents, including the source and type of documents and other information.
3. We propose an efficient and secure bucket-based red–black tree (B-RBT) data structure, which is used to support the dynamic operation of user data, and which accomplishes the update of the data and the re-balancing of the structure update in a constant time to achieve the high efficiency of the data update operation.
4. The correctness and security of the proposed scheme are proved by a specific security analysis, which is compared and analyzed with other schemes in the experimental simulation, and the experimental results show that the proposed scheme is secure and efficient.

### Related work

Cloud data auditing has garnered increasing attention in recent years. Ateniese et al.^11^ first proposed a public audit cloud data auditing scheme based on RSA homomorphic tagging technology. This scheme enables the remote verification of cloud data integrity by randomly selecting a subset of data blocks for auditing. Yang et al.^12^ proposed an auditing protocol with privacy preserving. This protocol combines data homomorphic authentication tags with the random masking approach. The combination of these techniques assures that a third-party auditor cannot access the user's private information while verifying integrity. Li et al.^13^ and Zheng et al.^14^ proposed approaches that aim to tackle the issue of privacy preservation in cloud data. Ping et al.^15^ proposed a cloud data auditing approach utilizing random sampling to verify data integrity. However, this method is limited to queries and does not support dynamic updates of cloud data. Yu et al.^16^ proposed an attribute-based cloud data auditing scheme, where users can define custom attribute sets and designate authorized third-party auditors to inspect the integrity of outsourced data. Jalil et al.^17^ has presented a public auditing scheme that utilizes BLS signatures for cloud data. This scheme effectively achieves public auditing goals while preserving data privacy. Nevertheless, the scheme's efficacy is strongly dependent on the PKI, necessitating a more intricate approach to certificate management. Ji et al.^18^ proposed an identity-based auditing scheme that effectively addresses the challenges related to certificate management in the context of PKI.

For delegated outsourced integrity auditing of data, Guo et al.^19^ introduced a novel approach for dynamic provable data possession, wherein the burden of frequent auditing tasks is shifted to an external auditor. This approach aims to alleviate the validation overhead experienced by the client. Additionally, the proposed scheme incorporates a secure auditor responsible for verifying the integrity of outsourced files. Notably, the auditor is not granted access to information about the user's files. Yang et al.^20^ proposed a proof-of-storage approach that allows for delegation and supports the involvement of third-party auditors. In the scenario that the designated auditor is not accessible, it is possible to substitute the auditor at any given moment, hence allowing for the appointment of a new auditor to conduct data integrity verification. Rao et al.^21^ introduced a novel dynamic auditing approach to outsourcing to mitigate the presence of dishonest entities and conflicts while enabling the verifiable dynamic update of outsourced data. Zhang et al.^22^ introduced an approach for data outsourcing with public integrity verification based on identification, wherein the original data owner can delegate an proxy to produce the data's signature and afterward outsource it to a cloud server. However, it is essential to note that none of the above schemes support a regulated delegated data outsourcing mechanism.

In response to the data update question, Thangavel et al.^23^ proposed a cloud storage auditing scheme based on Ternary Hash Tree (THT) that supports dynamic updating of data. This scheme enhances the efficiency of updating ternary trees compared to binary trees. Zou et al.^24^ mentioned a public auditing scheme that implements the Ranked-based Merkle Hash Tree (RMHT) to enable the auditing of secondary file blocks. Hariharasitaraman et al.^25^ introduced a novel public authentication approach that uses a Position aware Merkle tree (PMT). This scheme incorporates a ternary tuple scheme and exhibits strong resilience in offering authentication and data integrity services. Li et al.^26^ constructed a certificate-less verifiable data ownership mechanism that demonstrates efficiency. Additionally, He utilized the Dynamic Hash Table (DHT) to facilitate data updates and provide data privacy protection. Peng et al.^27^ proposed a cloud storage auditing scheme based on Multi-RepMlica Position-Aware Merkel Tree (MR-PMT), which can effectively verify the integrity of multi-replica data. However, the effectiveness of the auditing process is compromised when the size of replica files exceeds a certain threshold. Rao et al.^21^ presented the Batch-Leaves-Authenticated Merkle Hash Tree (BLA-MHT), which allows batch-authenticate of multiple leaf nodes and their corresponding indices while demonstrating resistance against replacement assaults. In Table 1, we present a comparative analysis of our proposed scheme and several relevant schemes, focusing on controlled data outsourcing, certificate management, source data auditing, and dynamic updates. Source data auditing refers to the process of auditing and verifying the source files. In our research, we propose a reliable source file auditing mechanism to ensure the consistency and integrity of the source files during data outsourcing. Theoretical analysis and experimental results have substantiated the inherent resilience and security characteristics of the proposed scheme, while maintaining performance levels without discernible degradation.

**Table 1:** The comparison of different program functions.

Schemes	Controlled outsourcing	Certificate management	Source data auditing	Dynamic update
Ref^5^	N	Y	N	Y
Ref^6^	N	N	N	N
Ref^7^	N	N	N	N
Ref^9^	N	N	N	N
Ref^13^	N	N	N	N
Ref^17^	N	Y	N	Y
Ref^19^	N	N	N	Y
Ref^21^	N	N	N	Y
Ref^24^	N	N	N	Y
Our scheme	Y	N	Y	Y

*Organization* The subsequent sections of the paper are structured in the following manner: “Preliminaries” section provides a comprehensive overview of the procedures that are necessary for the execution of this research study. A comprehensive account of the technique employed in this study is provided in “Method” section. “Security analysis” section provides a comprehensive security analysis of the proposed scheme. “Performance analysis” section provides a comprehensive evaluation of the proposed scheme performance. “Conclusion” section summarizes the full article.

## Preliminaries

### Bilinear mapping

A bilinear mapping^28^ is defined as follows: Given two multiplicative cyclic groups $G_{1}$ and $G_{2}$ of order *p*, where *p* is a large prime number, $g$ is a randomly chosen generator of group $G_{1}$. The bilinear pairing function $e:G_{1} \times G_{1} \to G_{2}$ has the following three features:

1. Bilinearity: For all $u,v \in G_{1}$ and $x,y \in {\mathbb{Z}}_{p}^{*}$, there exists $e(u^{x} ,v^{y} ) = e(u,v)^{xy}$;
2. Computability : For all $u,v \in G_{1}$, there exists an efficient algorithm to compute $e(u,v)$;
3. Non-degeneracy: For a generating element g of $G_{1}$ such that equation $e(g,g) \ne 1$ holds.

### Difficulty assumptions

Compute the Diffie-Hellman problem^7^: Given a multiplicative cyclic group $G_{1}$ of large prime order *p*, $g$ is a randomly generated element of $G_{1}$, and given $(g,g^{a} ,g^{b} ) \in G_{1}$, any probabilistic polynomial time algorithm $\Lambda$ is difficult to compute $g^{ab}$ in the unknown $a,b \in {\mathbb{Z}}_{p}^{*}$, i.e.,

$$ AdvCDH_{\Lambda } = \Pr [\Lambda (g,g^{a} ,g^{b} ) = g^{ab} ,a,b \in {\mathbb{Z}}_{p}^{*} ] \le \varepsilon $$

DL problem^29^: Given a multiplicative cyclic group $G_{1}$ of large prime order *p*, $g$ is a randomly generated element of $G_{1}$, and given $(g,g^{a} ) \in G_{1}$, it is hard for any probabilistic polynomial time algorithm $\Lambda$ to compute $a \in {\mathbb{Z}}_{p}^{*}$, i.e.,

$$ AdvDL_{\Lambda } = \Pr [\Lambda (g,g^{a} ) = a,a \in {\mathbb{Z}}_{p}^{*} ] \le \varepsilon $$

### Data structure

The red–black tree (RBT)^30^ is a self-balancing binary lookup tree where each node possesses an additional attribute indicating its color, which can be red or black. It maintains the balance of the tree by rotating or recoloring the nodes. During dynamic update operations, it has a time complexity of $O(1)$ for insertion and deletion operations and a worst-case time complexity of $O(\log n)$ for lookup. The properties of the red–black tree make an RBT of *n* nodes always maintain the height of $\log n$.

**Figure 1:**
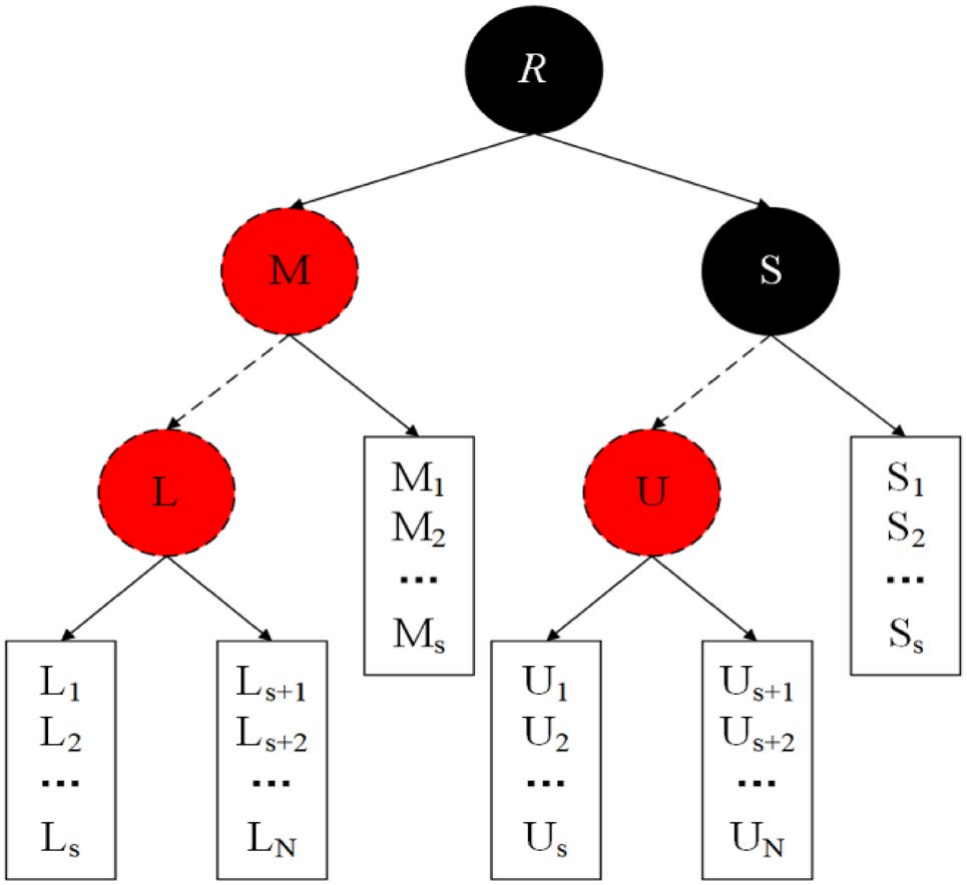
B-RBT data structure.

Bucket-based red–black tree (B-RBT) is a balanced search tree that can reach a rebalanced state of the tree in constant time in the worst case. Precisely, instead of storing a single key composition in the leaf nodes of the search tree, the structure consists of buckets storing an ordered list consisting of multiple keys in each leaf. The B-RBT proposed in this scheme does not require a global reconstruction technique, and for the deletion of nodes in the tree, there is no need for global reconstruction as in the case of a traditional red–black tree. As a result, the structure requires less space and time. The rules of the B-RBT structure are as follows:(1) each node has a color, red for red nodes and black for black nodes; (2) the root node is black; (3) each leaf node is a black bucket; and (4) if a node is red and the parent node is red as well, its children are all black, i.e., all paths where at most two consecutive red nodes exist; (5) the number of black nodes on each path starting from any node to its leaf node differs by at most 1. The B-RBT structure is shown in Fig. 1, where each node has an extra bit to indicate its color, and each node has the values of five attributes consisting of multiple keys for buckets, left child pointers, right child pointers, parent pointers, and color bits. The structure inserts elements into buckets instead of internal tree nodes, and each tree node stores a path value that is less than or equal to the key stored in the bucket of its right child tree and greater than the key of the bucket in its left child tree. Furthermore, the buckets are split when a single bucket increases to a specific value and merge when two neighboring buckets decrease to a specific value. Each bucket contains a header that attaches the bucket to a tree node and stores additional information about it, such as its size. For storing the size of the buckets, our scheme preserves a bucket threshold consistent with that proposed by Elmasry et al.^31^, i.e., the number of keys in each bucket must be in the range of 0.5–2 H. The splitting operation may result in the need to add a new node to the tree and may break the equilibrium state of the original B-RBT. Our goal is to maintain pointers to the first, middle, and last list nodes in the header of each bucket so that each update operation invokes a repair procedure and fixes it before the next split or merge violates the tree's equilibrium state, guaranteeing the correct setup of these pointers until then.

## Method

### System model

**Figure 2:**
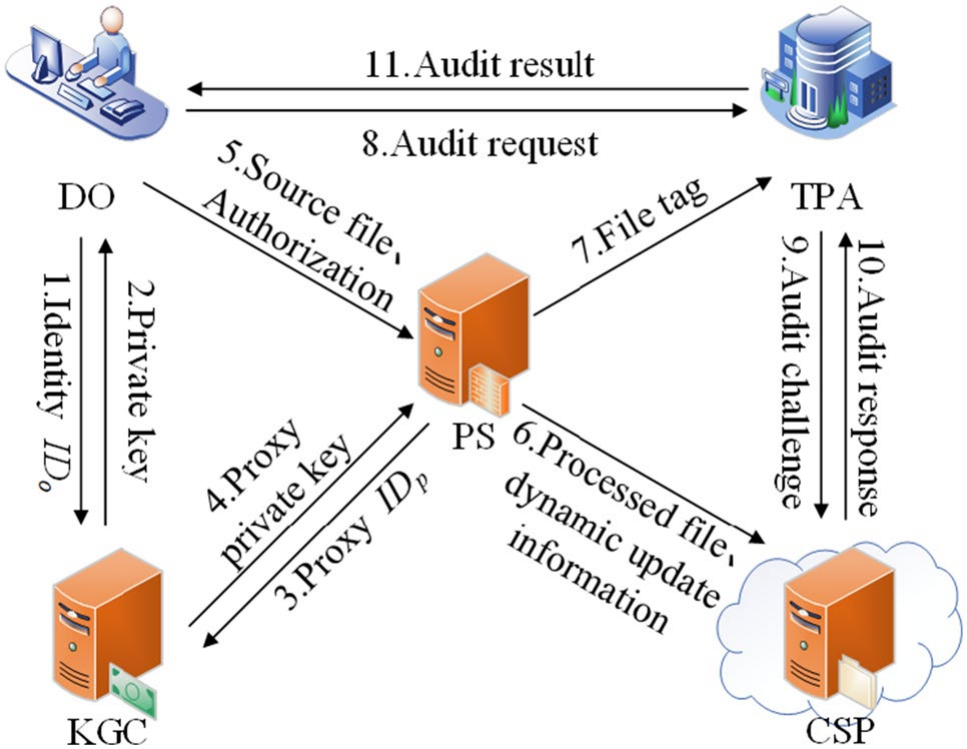
System model.

The scheme proposed in this paper contains a total of five entities: DO, KGC, PS, TPA and CSP, and the system model is shown in Fig. 2. Our proposed system model provides fine-grained control and authorization mechanisms to ensure data integrity with efficient dynamic data updating and source file auditing capabilities.

DO is the data owner, which stores the data in the cloud. CSP provides a powerful storage service for DO. PS is the proxy service provider where DO authorizes a PS to upload the data stored in CSP. DO blinds the data before uploading it to the PS. Blinding is a data processing technique aimed at concealing or protecting sensitive information while maintaining the utility of the data. In our research, the primary purpose of employing blinding techniques is to safeguard the privacy and confidentiality of the data, preventing unauthorized access and leakage. KGC generates private keys for DO and PS. In our system model, DO and KGC are trusted entities. CSP belongs to the category of incompletely trusted entities, which is trustworthy in data privacy but untrustworthy in data integrity; it may damage and tamper with DO's data. TPA belongs to the category of semi-trustworthy entities, which is trustworthy in terms of integrity verification but untrustworthy in terms of data privacy; it will fulfill the auditing tasks of DO as required, but it will be curious about the DO's data content is curious.

### Threat models

The proposed scheme faces three main types of attacks:

1. A malicious PS can mimic a DO or another authorized PS or abuse the DO's authorization delegation by processing the DO's files and uploading them to CSP storage.
2. Due to hardware failure or self-interest, a malicious CSP may delete or modify the DO's files, especially those accessed less frequently.
3. During the data outsourcing phase, there is a possibility that the PS or CSP may steal the DO's data. Semi-trustworthy TPA are unreliable in terms of data privacy and show curiosity about the content of data outsourced by DOs during the audit process.

### Security goals

Considering that the scheme may be subject to the above attacks, our scheme aims to achieve the following security objectives:

1. *Controllable delegation* Proofs of authorization generated by the DO can only be used by the specified PS to outsource specified files. Even authorized PS cannot misuse authorization proofs to outsource unspecified files, and multiple PSs cannot collude to infer new authorization proofs for outsourced unspecified files.
2. *Audit correctness* Means that only data evidence and tag evidence generated by the CSP is valid simultaneously to pass the TPA validation.
3. *Privacy preservation* In order to protect the sensitive information of the outsourced data, it is necessary to blind the data during the data outsourcing phase to protect the privacy and security of the data. During the audit process, TPA cannot retrieve the user's data information.

### Overview of the proposed scheme

The identity-based controlled delegated outsourced data integrity auditing scheme proposed in this paper consists of three phases (setup phase, auditing phase, and dynamic updating phase), and the setup and auditing phases contain the following eight polynomial time algorithms($SysSetup$, $KeyGen$,$DeleGen$,$DataBlind$,$DataOutsourcing$,$ChalGen$, $ProofGen$ and $ProofVerify$).

#### Setup phase

1. $SysSetup(1^{\kappa } ) \to (SysPara,msk)$. System setup algorithm. The KGC executes this algorithm and inputs the system security parameter $\kappa$, outputs the system global parameter $SysPara$, and the KGC's master key $msk$.
2. $KeyGen(SysPara,msk,ID_{i} ) \to sk_{i}$. Key generation algorithm. KGC executes this algorithm and takes as input the global parameters $SysPara$, master key $msk$, and identity $ID_{i}$ and outputs the corresponding key $sk_{i}$.
3. $DeleGen(SysPara,ID_{o} ,sk_{o} ,ID_{p} ) \to (\Phi ,\Gamma )$. Delegated authorization proof generation algorithm. The DO executes the algorithm and inputs the global parameter $SysPara$, the PS's identity $ID_{p}$, the DO's identity $ID_{o}$, and its key $sk_{o}$. It outputs the delegated proof of authorization information $Dele\_Info = (\Phi ,\Gamma )$, where $\Phi$ is the proof of authorization, and $\Gamma$ is the delegated proof.
4. $DataBlind(M,\pi ) \to M^{\prime}$. Data blinding algorithm. The plaintext data *M* and the blinding factor $\pi$ are used as input, and the blinded data $M^{\prime}$ is output.
5. $DataOutsourcing(SysPara,Dele\_Info,sk_{p} ,M^{\prime}) \to (\tau ,M^{*} )$ Data Outsourcing Algorithm. The PS executes the algorithm, which inputs the global parameters $SysPara$, the delegated authorization certificate information $Dele\_Info$, the key $sk_{p}$ of the PS, and the blinded source data $M^{\prime}$ to be outsourced and outputs the corresponding file tag $\tau$ and the processed data M^*^.

#### Audit phase

1. $ChalGen(SysPara,\tau ) \to chal$. Audit challenge generation algorithm. The TPA executes this algorithm by inputting the global parameters $SysPara$ and the file tag $\tau$. After the file tag $\tau$ passes the legitimacy verification by the TPA, it outputs the audit challenge $chal$.
2. $ProofGen(SysPara,chal) \to proof$ Proof generation algorithm. The CSP executes the algorithm. Inputs the global parameters $SysPara$ and the audit challenge $chal$ and outputs the audit challenge proof $proof = (\delta ,\eta )$, where $\delta$ is the tag evidence, and $\eta$ is the data evidence.
3. $ProofVerify(SysPara,\tau ,proof) \to (True/False)$. Proof validation algorithm. The TPA executes this algorithm by inputting the global parameter $SysPara$, audit challenge proof $proof$ and the file tag $\tau$, and outputting *True* when the evidence validation passes, otherwise outputting *False*.

### Security definitions

This subsection presents formal security definitions to fulfill the above security goals. Two probabilistic polynomial-time adversaries,$A_{1}$ and $A_{2}$,are used to simulate a malicious PS and a malicious CSP, respectively. $A_{1}$ has the capability to simulate collusion in order to forge or misuse the authorization of DO, and it can also simulate the CSP to modify the stored files of DO without being detected.

**Game 1:** We have defined a security game against malicious PS based on the concept of probabilistic polynomial time. The adversary $A_{1}$ plays the following game with the challenger $C$:

**Setup:** The challenger $C$ runs the system setup algorithm $SysSetup(1^{\kappa } ) \to (SysPara,msk)$ to obtain the global parameters $SysPara$ and the master key $msk$ and sends $SysPara$ to adversary $A_{1}$.

**Queries:** Adversary $A_{1}$ performs multiple polynomial queries to the challenger $C$, and challenger $C$ responds to the polynomial queries of the adversary $A_{1}$ as follows:

1. Extraction query: The adversary $A_{1}$ performs a private key extraction query on all identifiers $ID_{i}$. Challenger $C$ computes the private key $sk_{i}$ of $ID_{i}$ and sends it to $A_{1}$.
2. Delegation query: Adversary $A_{1}$ submits proof of delegation $\Phi$ to challenger $C$. If DO's private key $sk_{o}$ has yet to be queried before, challenger $C$ first generates DO's private key $sk_{o}$. Then challenger $C$ responds with delegation proof $\Phi$.
3. File processing query: Adversary $A_{1}$ submits the proof of authorization $(\Phi ,\Gamma )$ to the challenger $C$ in this query. If the PS's private key $sk_{p}$ and proof of authorization $\Phi$ have yet to be queried, challenger $C$ generates them first. Then challenger $C$ responds with the processed file $M^{*}$.

**Output:** Eventually, adversary $A_{1}$ generates a processed file $\widehat{{M^{*} }}$ under the legal authorization $\widehat{\Phi }$. Adversary $A_{1}$ wins the game if the following conditions are met:

1. The adversary $A_{1}$ obtains its private key without extracting a query from $ID_{o}$;
2. Adversary $A_{1}$ has failed to conduct a proxy search on the certificate of authority $\widehat{\Phi }$;
3. Adversary $A_{1}$ did not perform a file processing query involving proof of authorization $\widehat{\Phi }$;
4. Proof of delegation $\widehat{\Gamma }$ is $\widehat{{ID_{o} }}$ valid for $\widehat{{ID_{p} }}$.

#### **Definition 1**

An identity-based CDODIA scheme is secure against adaptive mimicry and abuse of delegated proofs if any probabilistic polynomial-time adversary $A_{1}$ and challenger $C$ win the above game with negligible probability only.

**Game 2:** We define security games against malicious CSP with probabilistic polynomial time adversaries $A_{2}$. Play the following game with challengers $C$:

**Setup:** Challenger $C$ runs the system setup algorithm $SysSetup(1^{\kappa } ) \to (SysPara,msk)$ to obtain the global parameters $SysPara$ and the master key $msk$ and sends $SysPara$ to adversary $A_{2}$.

**Queries:** Challenger $C$ adaptively interacts with adversary $A_{2}$ to enforce the integrity auditing protocol. Here adversary $A_{2}$ plays the role of a validator, which can respond to any integrity challenge initiated by the challenger $C$.

1. Challenge generation query: Challenger $C$ generates a challenge response for a particular data file and sends it to $A_{2}$.
2. Challenge response query: Adversary $A_{2}$ generates evidence as a challenge-response based on processed file.
3. Challenge validation query: Challenger $C$ validates its challenge-response evidence and returns the validation results to the adversary $A_{2}$.

**Output:** Finally, challenger $C$ and adversary $A_{2}$ complete the last round of the integrity audit protocol. Challenger $C$ sends an audit challenge $\widehat{chal}$ against a processed file $\widehat{{M^{*} }}$, and adversary $A_{2}$ generates evidence $\widehat{proof}$ as a challenge-response. Assume that the file $\widehat{{M^{*} }}$ stored in the CSP has been corrupted or modified and that the audit challenge $\widehat{chal}$ contains the corrupted or modified data. Suppose evidence $\widehat{proof}$ contains a valid tuple $(\widehat{\vartheta },\{ \widehat{{\eta_{j} }}\}_{1 \le j \le c} )$ for the audit challenge $\widehat{chal}$ and the processed file $\widehat{{M^{*} }}$, and the tuple is not the same as the audit challenge $chal$ and the correctly maintained file $\widehat{{M^{*} }}$. In that case, it is stated that adversary $A_{2}$ wins the game.

#### **Definition 2**

An identity-based CDODIA scheme is secure against data modification attacks if any probabilistic polynomial-time adversary $A_{2}$ wins the above game only with negligible probability.

### Detailed description of the proposed scheme

**Figure 3:**
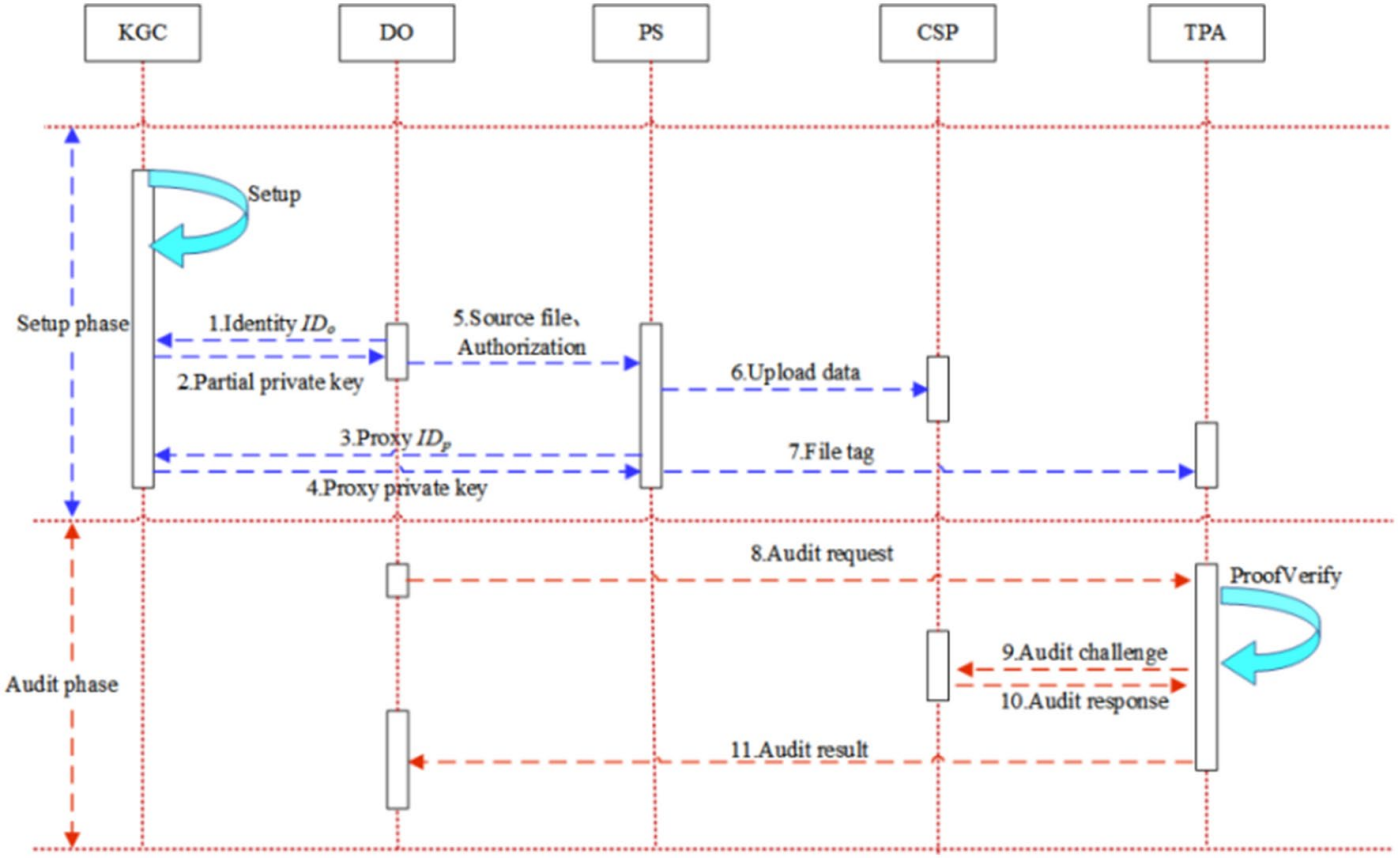
Schematic diagram of the audit process.

In this section, the three stages of the proposed scheme are described in detail, and the audit process is illustrated in Fig. 3.

#### Detailed description of setup phase

$$ SysSetup(1^{\kappa } ) \to (SysPara,msk) $$

KGC chooses two multiplicative cyclic groups $G_{1}$ and $G_{2}$ of large prime order *p*, based on the security parameter $\kappa$*. g* is a randomly generated element,$g \in G_{1}$. Selecting a bilinear pairing function $e:G_{1} \times G_{1} \to G_{2}$. KGC randomly selects the integer $x \in {\mathbb{Z}}_{p}^{*}$ and the element $(g_{1} ,\{ \mu_{i} \}_{0 \le i \le l} ,\{ v_{i} \}_{0 \le i \le \ell } ,\{ u_{i} \}_{0 \le i \le n} ) \in G_{1}$, and computes $y = g^{x}$, the master key $msk = g_{1}^{x}$. Three collision-resistant hash functions are chosen: $H_{1} :\{ 0,1\}^{*} \to \{ 0,1\}^{l}$,$H_{2} :\{ 0,1\}^{*} \to \{ 0,1\}^{\ell }$,and $H_{3} :\{ 0,1\}^{*} \to G_{1}$.

The final system parameters $SysPara = \{ G_{1} ,G_{2} ,e,p,g_{1} ,y,H_{1} ,H_{2} ,H_{3} ,\{ \mu_{i} \}_{0 \le i \le l} ,\{ v_{i} \}_{0 \le i \le \ell } ,\{ u_{i} \}_{0 \le i \le n} \}$ are published, and the master key $msk$ is kept secretly by KGC itself.

$$ KeyGen(SysPara,msk,ID_{i} ) \to sk_{i} $$

After receiving the identity $ID_{i}$ from DO or PS, KGC generates the private key $sk_{i}$ for DO and PS according to its own master key $msk$, which is as follows: first, KGC calculates

1 $$ \widetilde{u}_{i} = ({\text{u}}_{i,1} ,{\text{u}}_{{i,{2}}} , \ldots ,{\text{u}}_{i,l} ) \leftarrow H_{1} (ID_{i} ) $$

Then, it randomly selects $\sigma_{i} \in {\mathbb{Z}}_{p}^{*}$ and generates the private key $sk_{i} = (sk_{i,1} ,sk_{i,2} )$, where

2 $$ sk_{i,1} = msk \cdot (\mu_{0} \cdot \prod\limits_{j = 1}^{l} {\mu_{j}^{{{\text{u}}_{i,j} }} } )^{{\sigma_{i} }} $$

and

3 $$ sk_{i,2} = g^{{\sigma_{i} }} $$

Finally, send $sk_{i}$ to DO or PS. DO, and PS can verify whether their private key $sk_{i}$ is legitimate by calculating $\widetilde{u}_{i}$ and checking the equation

4 $$ e(sk_{i,1} ,g)\mathop = \limits^{?} e(y,g_{1} ) \cdot e \left(\mu_{0} \cdot \prod\limits_{j = 1}^{l} {\mu_{j}^{{{\text{u}}_{i,j} }} } ,sk_{i,2}\right ) $$

If the equation is valid, they accept the private key $sk_{i}$ and reject it otherwise.

$$ DeleGen(SysPara,ID_{o} ,sk_{o} ,ID_{p} ) \to (\Phi ,\Gamma ) $$

To authorize a legitimate proxy PS, the DO generates an authorization proof $\Phi$ containing the DO's identity $ID_{o}$ and the proxy PS's identity $ID_{p}$. The authorization proof $\Phi$ may also contain other information related to the source data *M*, such as the time of delegation, file type, etc., i.e., $\Phi = (ID_{o} ||ID_{p} ||TimeStamp||Type)$. Based on the system parameter $SysPara$, the DO first calculates

5 $$ \widetilde{\varphi } = (\phi_{1} ,\phi_{2} , \ldots ,\phi_{\ell } ) \leftarrow H_{2} (\Phi ) $$

Then, it randomly selects $\sigma_{\phi } \in {\mathbb{Z}}_{p}^{*}$ and generates the proof of delegation $\Gamma = (\alpha ,\beta ,\gamma )$, where

$$\alpha = sk_{o,1} \cdot \left(v_{0} \cdot \prod\limits_{j = 1}^{\ell } {v_{j}^{{\phi_{j} }} }\right )^{{\sigma_{\phi } }}, \;\; \beta = sk_{o,2}, \;\; \gamma = g^{{\sigma_{\phi } }}.$$

Finally, the proof of delegation $(\Phi ,\Gamma )$ is sent to the proxy PS. After receiving the information of the proof of delegation from the DO, the PS can verify the legitimacy of the delegation information by calculating $\widetilde{u}_{o}$ and $\widetilde{\varphi }$ as well as checking the equation

6 $$ e(\alpha ,g)\mathop = \limits^{?} e(y,g_{1} ) \cdot e(sk_{o,1}^{{\frac{1}{{\sigma_{o} }}}} ,\beta ) \cdot e \left (v_{0} \cdot \prod\limits_{j = 1}^{\ell } {v_{j}^{{\phi_{j} }} } ,\gamma \right) $$

If the equation is valid, the PS receives the delegation from the DO; otherwise the delegation request is rejected.

$$ DataBlind(M,\pi ) \to M^{\prime} $$

Given an outsourced data source file $M \in \{ 0,1\}^{*}$ with legitimate proof-of-authorization information, the DO divides the file into *n* data blocks,$M = \{ m_{1} ,m_{2} , \ldots ,m_{n} \}$. $\pi \in {\mathbb{Z}}_{p}^{*}$ is randomly selected as the blinding factor, and each blinded data block $m_{i} ^{\prime} = (m_{i} ||i) + \pi$ is computed. Finally, the blinded file $M^{\prime}$ is sent to the PS.

$$ DataOutsourcing(SysPara,Dele\_Info,sk_{p} ,M^{\prime}) \to (\tau ,M^{*} ) $$

After receiving the blinded data from the DO, firstly, the PS divides the blinded data $M^{\prime}$ into *c *$(1 \le c \le n)$ sectors, i.e., $M^{\prime} = \{ m_{i,j} ^{\prime}\}_{r \times c}$. Second, PS randomly selects the source file identifier $F_{id} \in {\mathbb{Z}}_{p}^{*}$ and the random value $\sigma_{F} \in {\mathbb{Z}}_{p}^{*}$ and computes $v_{F} = g^{{\sigma_{F} }}$ and sets $\tau_{0} = \Phi ||F_{id} ||v_{F} ||r$. PS selects the signature algorithm $PS.Sign = (KeyGen,Sign,Verify)$^32^ to sign $\tau_{0}$ and generates the source file tag $\tau = \tau_{0} ||PS.Sign.Sign(\tau_{0} ,ssk)||spk$, where the signed public–private key pair $(ssk,spk)$ is generated by the algorithm $PS.Sign.KeyGen(1^{\kappa } )$. Then PS uses its private key $sk_{p}$ to sign the data block

7 $$ \vartheta_{i} = sk_{p,1} \cdot \left(H_{3} (\Psi ) \cdot \prod\limits_{j = 1}^{c} {u_{j}^{{m_{i,j} ^{\prime}}} } \right)^{{\sigma_{F} }} $$

where $\Psi = \Phi ||F_{id} ||i$. Finally, the processed source files $M^{*} = (M^{\prime},F_{id} ,\Gamma ,\{ \vartheta_{i} \}_{1 \le i \le c} )$ and $(\tau ,\{ \vartheta_{i} \}_{1 \le i \le c} )$ are sent to CSP and TPA, respectively, and $M^{*}$ and $\{ \vartheta_{i} \}_{1 \le i \le c}$ are deleted locally.

#### Detailed description of audit phase

In this phase, the TPA performs integrity auditing of the cloud data from time to time, which contains the following algorithms:

$$ ChalGen(SysPara,\tau ) \to chal $$

Before the integrity audit begins, the TPA runs the algorithm $PS.Sign.Verify(\tau_{0} ,spk)$ to verify the legitimacy of the file tag $\tau$. If the validation fails, the audit request is rejected; if it passes the validation, TPA randomly selects a non-empty subset $S_{F}$ from the set $[1,r]$ and randomly selects $s_{i} \in {\mathbb{Z}}_{p}^{*}$ to generate the audit challenge $chal = (i,s_{i} )_{{i \in S_{F} }}$, and finally sends $(chal,F_{id} )$ to the CSP.

$$ ProofGen(SysPara,chal) \to proof $$

Upon receiving the audit challenge from the TPA, the CSP locates the files $M^{*}$ to be integrity audited based on $(chal,F_{id} )$ and calculates the tag proof

8 $$ \vartheta = \prod\limits_{{i \in S_{F} }} {\vartheta_{i}^{{s_{i} }} } $$

and data proof

9 $$ \eta_{j} = \sum\limits_{{i \in S_{F} }} {s_{i} \cdot m^{\prime}_{i,j} } (j \in [1,c]) $$

Finally,$proof = (\vartheta ,\{ \eta_{j} \}_{1 \le j \le c} )$ is sent to the TPA along with the delegation $\Gamma$.

$$ ProofVerify(SysPara,\tau ,proof) \to (True/False) $$

After receiving *the proof*, the TPA calculates $(\widetilde{u}_{o} ,\widetilde{u}_{p} ,\widetilde{\varphi })$ based on the system parameters $SysPara$ and the file tag $\tau$. Then TPA verifies the legitimacy of the delegation $\Gamma$ according to Eq. (6); if the equation is valid, it means that the PS has a proof of legitimate authorization. Finally, TPA performs an integrity audit by checking equation

10 $$ \begin{gathered} e(\vartheta ,g)\mathop = \limits^{?} (e(y,g_{1} ) \cdot e(sk_{p,1}^{{\frac{1}{{\sigma_{p} }}}} ,sk_{p,2} ))^{{\sum\limits_{{i \in S_{F} }} {s_{i} } }} \hfill \\ \cdot e \left(\prod\limits_{{i \in S_{F} }} {H_{3} (\Psi )^{{s_{i} }} \cdot \prod\limits_{j = 1}^{c} {u_{j}^{{\eta_{j} }} } } ,v_{F} \right) \hfill \\ \end{gathered} $$

If the equation is valid and outputs *True*, the file stored in the CSP is complete; otherwise, if it outputs *False*, the file is incomplete.

#### Detailed description of dynamic update phase

The B-RBT structure proposed in this scheme can effectively support dynamic updating of data (modification, insertion and deletion) in the following process:

1. Data insertion process: when DO needs to insert a new data block after a certain data block, DO generates the corresponding dynamic updated information $Update\_Info\_PS = (Insert,i,Dele\_Info^{\prime},M^{\prime})$ and sends it to PS, where *Insert* is the insert command, *i* is the location to be inserted,$M^{\prime}$ is the newly inserted data, and $Dele\_Info^{\prime}$ is the delegated authorization proof information of the new data $M^{\prime}$. After receiving the dynamic updated information, PS generates the new updated information $Update\_Info\_TPA = (Insert,i,M^{^{\prime}*} )$ after processing the new data $M^{\prime}$, and sends the new updated information to TPA. The TPA receives the update information and stores the signature of the data block to be inserted in the B-RBT data structure, inserts a new list node at the specified position of the specified bucket *B*, and calls the repair process of bucket *B*. The TPA receives the updated information and stores the signature of the data block to be inserted in the B-RBT data structure. When the bucket exceeds the maximum threshold, if the repair pointer *P*_*K*_ has not yet reached the root node *R*, the repair process of *B* is called repeatedly until PK reaches *R*. Then bucket *B* is split into two buckets $\{ B_{1} ,B_{2} \}$, and the new parent node *B*_12_ of $\{ B_{1} ,B_{2} \}$ is added to the tree in the form of a red node, and the pointers of the two new buckets are made to point to the newly inserted nodes. Finally, the repair process is called again for one of the two buckets to complete the signature storage and update these data records in the CSP. Figure 4a shows that after inserting data into bucket *S* in Fig. 1, the capacity of its bucket exceeds over the maximum threshold, so bucket *S* is divided into two buckets, node *K* is used to manage the two new buckets and node *K* is labeled as red.

**Figure 4:**
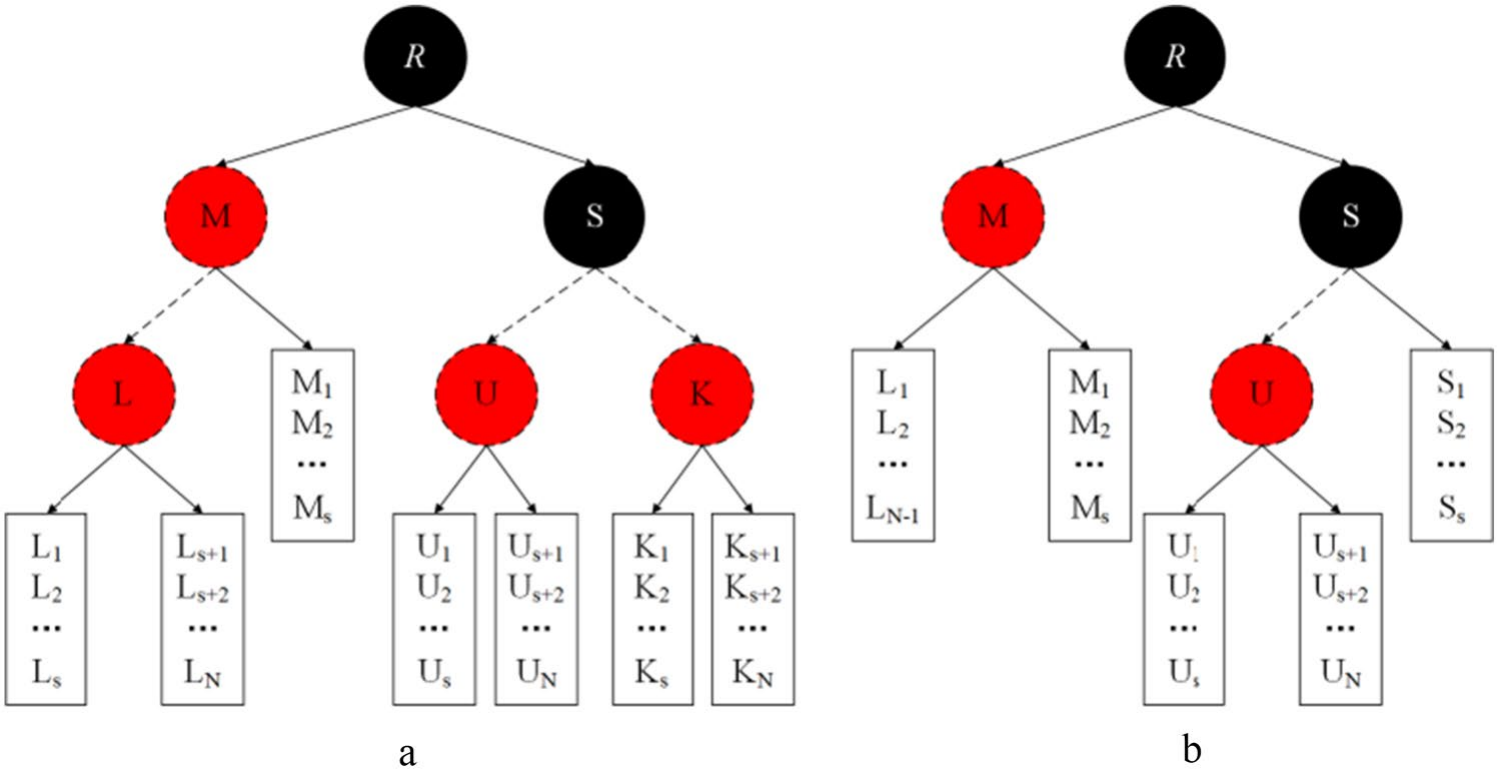
B-RBT structure update.
2. Data deletion process: When the DO needs to delete a block of data, DO generates the corresponding dynamic updated information $Update\_Info\_PS = (Delete,i,Dele\_Info^{ \bullet } )$ and sends it to PS, where *Delete* is the delete command, *i* is the location of the deleted data block, and $Dele\_Info^{ \bullet }$ is the delegated authorization proof information of the required deleted data block. After receiving the dynamic update information, PS generates the new update information $Update\_Info\_TPA = (Delete,i)$ and sends the new update information to the TPA. TPA first finds the required list node from bucket *B*, finds the list nodes required for deletion, and invokes the repair process of bucket *B* twice. When the bucket is smaller than the minimum threshold if the repair pointer *P*_*K*_ has not yet reached the root node *R*, it repeats the repair process of *B* until *P*_*K*_ reaches *R*. If the size of the sibling bucket $B^{\prime}$ of bucket *B* exceeds the minimum threshold, *B* borrows a node from $B^{\prime}$ and calls the repair process twice for *B*. If the size of bucket *B*'s sibling bucket $B^{\prime}$ does not exceed the minimum threshold, *B* and $B^{\prime}$ are merged to form a new bucket $B^{\prime\prime}$, where the key of the right bucket is placed at the end of the left bucket, and the parent node of $B^{\prime\prime}$ is the grandfather node of the original *B* or $B^{\prime}$. If the deleted parent bucket is black, mark $B^{\prime\prime}$ double black to complete the data block signature deletion and update these data records in the CSP. Figure 4b shows the merging of bucket* L* and sibling bucket $L^{\prime}$ with node *M* when the size of bucket *L*'s sibling bucket $L^{\prime}$ will not exceed the minimum threshold after deleting the data of bucket *L* in Fig. 1.
3. Data modification process: The operation is stored in the CSP to correct certain information. The specific operation and insertion and deletion of the same can be viewed as the original data deletion operation, in the insertion of new data to complete.

## Security analysis

Suppose the entities in the scheme proposed in this paper are honest in executing the individual protocols as expected. In that case, key generation, delegation generation, and file processing can be audited correctly.

### **Theorem 1**

Privacy Preservation. PS, TPA or CSP cannot extract the DO's original data in the acquired data. In the data outsourcing phase, PS or CSP cannot extract the real data of DO from the blind data. In the auditing phase, TPA cannot obtain the real data of DO from the data signature.

### *Proof*

Data privacy security means that an adversary cannot obtain any original data information without the corresponding data decryption key. Firstly, in the data outsourcing phase, the blind data blocks $M^{\prime}$ and the blinding factors $\pi \in {\mathbb{Z}}_{p}^{*}$ are random functions generated by the DO randomly selecting the key seeds, and the blind data blocks are generated independently from each other, and thus the PS or the CSP wanting to extract the real data information M from them can be treated as a DL problem, and the probability of solving the DL problem can be negligible. Secondly, in the auditing phase, it can be verified by the following equation:

$$ \begin{aligned} \vartheta & = \prod\limits_{{i \in S_{F} }} {\vartheta_{i}^{{s_{i} }} } \hfill \\ & = \prod\limits_{{i \in S_{F} }} { \left (sk_{p,1} \cdot \left (H_{3} (\Psi ) \cdot \prod\limits_{j = 1}^{c} {u_{j}^{{m_{i,j} ^{\prime}}} } \right)^{{\sigma_{F} }} \right)^{{s_{i} }} } \hfill \\ & = \prod\limits_{{i \in S_{F} }} {\left((sk_{p,1} \cdot (H_{3} (\Psi ))^{{s_{i} }} \cdot \prod\limits_{j = 1}^{c} {u_{j}^{{\sum\limits_{{i \in S_{F} }} {s_{i} \cdot } m_{i,j} ^{\prime}}} } \right)^{{\sigma_{F} }} } \hfill \\ & = \prod\limits_{{i \in S_{F} }} {\left(sk_{p,1} \cdot (H_{3} (\Psi )\right)^{{s_{i} \cdot \sigma_{F} }} \cdot \left(\prod\limits_{j = 1}^{c} {u_{j}^{{\eta_{j} }} } \right)^{{\sigma_{F} }} } \hfill \\ \end{aligned} $$

From the above derivation it can be seen that if the values of $(sk_{p,1} \cdot H_{3} (\Psi ))^{{s_{i} }}$ and $g^{{\sigma_{F} }}$ are given, it is computationally infeasible to try to derive the value of $\prod\nolimits_{{i \in S_{F} }} {(sk_{p,1} \cdot (H_{3} (\Psi ))^{{s_{i} \cdot \sigma_{F} }} }$,and that $\prod\nolimits_{j = 1}^{c} {(u_{j}^{{\eta_{j} }} } )^{{\sigma_{F} }}$ can be viewed as blinded by $\prod\nolimits_{{i \in S_{F} }} {(sk_{p,1} \cdot (H_{3} (\Psi ))^{{s_{i} \cdot \sigma_{F} }} }$. Thus the TPA trying to extract the real data information from the tag proof $\vartheta$ can be viewed as a CDH problem, and the probability of solving the CDH problem is negligible.

### **Theorem 2**

In the setup phase, all DOs accept the private key generated by KGC, and PS accepts the delegation requests from all honest DOs. If the outsourced files designated for delegation are not corrupted or tampered with, then the evidence generated by CSP can be verified valid by TPA.

### *Proof*

From the previous section, the conditions for Eq. (4) to hold are obvious, and its correctness will not be proved in detail here. From Eq. (6),$\widetilde{u}_{o} = ({\text{u}}_{o,1} ,{\text{u}}_{o,2} , \ldots ,{\text{u}}_{o,l} )$, and $\widetilde{\varphi } = (\phi_{1} ,\phi_{2} , \ldots ,\phi_{\ell } )$ can be calculated by Eqs. (1) and (5). Therefore there are

$$ \begin{aligned} e \left(\alpha ,g\right) & = e \left(g_{1}^{x} \cdot  \left(\mu_{0} \cdot \prod\limits_{j = 1}^{l} {\mu_{j}^{{{\text{u}}_{o,j} }} } \right)^{{\sigma_{o} }} \cdot  \left(v_{0} \cdot \prod\limits_{j = 1}^{l} {v_{j}^{{\phi_{j} }} } \right)^{{\sigma_{\phi } }} ,g\right) \hfill \\ & = e \left(g_{1}^{x} ,g\right) \cdot e \left( \left(\mu_{0} \cdot \prod\limits_{j = 1}^{l} {\mu_{j}^{{{\text{u}}_{o,j} }} } \right)^{{\sigma_{o} }} ,g\right) \cdot e \left( \left(v_{0} \cdot \prod\limits_{j = 1}^{l} {v_{j}^{{\phi_{j} }} } \right)^{{\sigma_{\phi } }} ,g\right) \hfill \\ & = e \left(g_{1} ,g^{x} \right) \cdot e \left(\mu_{0} \cdot \prod\limits_{j = 1}^{l} {\mu_{j}^{{{\text{u}}_{o,j} }} } ,g^{{\sigma_{o} }} \right) \cdot e \left(v_{0} \cdot \prod\limits_{j = 1}^{l} {v_{j}^{{\phi_{j} }} } ,g^{{\sigma_{\phi } }} \right) \hfill \\ & = e \left(y,g_{1} \right) \cdot e \left(sk_{o,1}^{{\frac{1}{{\sigma_{o} }}}} ,\beta \right) \cdot e \left(v_{0} \cdot \prod\limits_{j = 1}^{\ell } {v_{j}^{{\phi_{j} }} } ,\gamma \right) \hfill \\ \end{aligned} $$

From the above equation, Eq. (6) holds.

The evidence *proof* returned by the CSP can be obtained from $\eta_{j} = \sum\nolimits_{{i \in S_{F} }} {s_{i} \cdot m_{i,j} ^{\prime}} (j \in [1,c])$,$\vartheta = \prod\nolimits_{{i \in S_{F} }} {\vartheta_{i}^{{s_{i} }} }$, where

$$ \begin{aligned} \vartheta & = \prod\limits_{{i \in S_{F} }} {\vartheta_{i}^{{s_{i} }} = } \prod\limits_{{i \in S_{f} }} {  \left(sk_{p,1} \cdot  \left(H_{3} (\Psi ) \cdot \prod\limits_{j = 1}^{c} {u_{j}^{{m_{i,j} ^{\prime}}} }\right )^{{\sigma_{F} }}\right )^{{s_{i} }} } \hfill \\  & = \prod\limits_{{i \in S_{f} }} {  \left(g_{1}^{x} \cdot \left(\mu_{0} \cdot \prod\limits_{j = 1}^{l} {\mu_{j}^{{{\text{u}}_{p,j} }} } \right)^{{\sigma_{p} }} \cdot  \left(H_{3} (\Psi ) \cdot \prod\limits_{j = 1}^{c} {u_{j}^{{m_{i,j} ^{\prime}}} } \right)^{{\sigma_{F} }}\right )^{{s_{i} }} } \hfill \\ & = \prod\limits_{{i \in S_{f} }} { (g_{1}^{x} )^{{s_{i} }} } \cdot \prod\limits_{{i \in S_{f} }} { \left(  \left(\mu_{0} \cdot \prod\limits_{j = 1}^{l} {\mu_{j}^{{{\text{u}}_{p,j} }} }\right )^{{\sigma_{p} }}\right )^{{s_{i} }} } \cdot \prod\limits_{{i \in S_{f} }} { \left( \left(H_{3} (\Psi ) \cdot \prod\limits_{j = 1}^{c} {u_{j}^{{m_{i,j} ^{\prime}}} } \right)^{{\sigma_{F} }}\right )^{{s_{i} }} } \hfill \\ &= (g_{1}^{x} )^{{\sum\limits_{{i \in S_{F} }} {s_{i} } }} \cdot \left( \left(\mu_{0} \cdot \prod\limits_{j = 1}^{l} {\mu_{j}^{{{\text{u}}_{p,j} }} } \right)^{{\sigma_{p} }}\right )^{{\sum\limits_{{i \in S_{F} }} {s_{i} } }} \cdot \left (\prod\limits_{{i \in S_{f} }} { \left(H_{3} (\Psi )^{{s_{i} }} \cdot \prod\limits_{j = 1}^{c} {u_{j}^{{\sum\limits_{{i \in S_{F} }} {s_{i} \cdot m_{i,j} ^{\prime}} }} } \right)^{{\sigma_{F} }}  }\right) \hfill \\ \end{aligned} $$

From Eq. (10), it follows that

$$ \begin{aligned} e(\vartheta ,g) & = e((g_{1}^{x} )^{{\sum\limits_{{i \in S_{F} }} {s_{i} } }} \cdot \left ( \left (\mu_{0} \cdot \prod\limits_{j = 1}^{l} {\mu_{j}^{{{\text{u}}_{p,j} }} } \right)^{{\sigma_{p} }} \right)^{{\sum\limits_{{i \in S_{F} }} {s_{i} } }} \hfill \\ & \quad \cdot \left (\prod\limits_{{i \in S_{f} }} { \left(H_{3} (\Psi )^{{s_{i} }} \cdot \prod\limits_{j = 1}^{c} {u_{j}^{{\sum\limits_{{i \in S_{F} }} {s_{i} \cdot m_{i,j} ^{\prime}} }} } \right)^{{\sigma_{F} }} } ,g \right) \hfill \\ & = e((g_{1}^{x} )^{{\sum\limits_{{i \in S_{F} }} {s_{i} } }} ,g) \cdot e \left ( \left ( \left(\mu_{0} \cdot \prod\limits_{j = 1}^{l} {\mu_{j}^{{{\text{u}}_{p,j} }} } \right)^{{\sigma_{p} }} \right)^{{\sum\limits_{{i \in S_{F} }} {s_{i} } }} ,g \right) \hfill \\ & \quad \cdot e  ( (\prod\limits_{{i \in S_{f} }} {(H_{3} (\Psi )^{{s_{i} }} \cdot \prod\limits_{j = 1}^{c} {u_{j}^{{\sum\limits_{{i \in S_{F} }} {s_{i} \cdot m_{i,j} ^{\prime}} }} } )^{{\sigma_{F} }} } ,g ) \hfill \\ & = e(g_{1} ,g^{x} )^{{\sum\limits_{{i \in S_{F} }} {s_{i} } }} \cdot e \left(\mu_{0} \cdot \prod\limits_{j = 1}^{l} {\mu_{j}^{{{\text{u}}_{p,j} }} } ,g^{{\sigma_{p} }} \right)^{{\sum\limits_{{i \in S_{F} }} {s_{i} } }} \hfill \\ & \quad \cdot e \left(\prod\limits_{{i \in S_{f} }} {H_{3} (\Psi )^{{s_{i} }} \cdot \prod\limits_{j = 1}^{c} {u_{j}^{{\eta_{j} }} } } ,g^{{\sigma_{F} }} \right) \hfill \\ & = (e(y,g_{1} ) \cdot e(sk_{p,1}^{{\frac{1}{{\sigma_{p} }}}} ,sk_{p,2} ))^{{\sum\limits_{{i \in S_{F} }} {s_{i} } }} \cdot e \left(\prod\limits_{{i \in S_{F} }} {H_{3} (\Psi )^{{s_{i} }} \cdot \prod\limits_{j = 1}^{c} {u_{j}^{{\eta_{j} }} } } ,v_{F} \right) \hfill \\ \end{aligned} $$

From the above proof, Eq. (10) holds.

### **Theorem 3**

If the CDH difficulty assumption problem holds and the signature scheme $PS.Sign = (KeyGen,Sign,Verify)$ employed by PS is computationally secure, then our scheme is secure and capable of withstanding adaptive attacks and abuse of delegation attacks, i.e., no cloud client or malicious CSP can collude with PS to construct a new delegation proof and use this delegation proof to outsource files.

### *Proof*

Suppose that adversary $A$ generates with probability $\varepsilon$ a processed file containing a forged delegation. Then, given a multiplicative cyclic group $G_{1}$ with element $g$ and a CDH difficulty assuming $(g,g^{\chi } ,\zeta )$, challenger $C$ and the adversary $A$ interact adaptively to compute $\zeta^{\chi }$.

**Setup:** Challenger $C$ sets up $l_{u} = 2(q_{e} + q_{s} )$,$l_{w} = 2q_{s}$, where $q_{e}$ and $q_{s}$ denote the total number of extracted queries and delegated queries, respectively. Assume that we have $l_{u} (l + 1) < p$ and $l_{w} (\ell + 1) < p$. The challenger $C$ picks random values $k_{u} \in [1,l]$, $k_{w} \in [1,\ell ]$,$x^{\prime} \in {\mathbb{Z}}_{{l_{u} }}^{*}$,$z^{\prime} \in {\mathbb{Z}}_{{l_{w} }}^{*}$ ,$y^{\prime},\varpi ^{\prime} \in {\mathbb{Z}}_{p}^{*}$,$x_{i} \in {\mathbb{Z}}_{{l_{u} }}^{*} (1 \le i \le l)$,$y_{i} \in {\mathbb{Z}}_{p}^{*} (1 \le i \le l)$,$z_{j} \in {\mathbb{Z}}_{{l_{w} }}^{*} (1 \le j \le \ell )$,$\varpi_{j} \in {\mathbb{Z}}_{p}^{*} (1 \le j \le \ell )$,$\pi_{j} ,\omega_{j} \in {\mathbb{Z}}_{p}^{*} (1 \le j \le c)$.

Next, the challenger $C$ generates parameters as follows:$\mu_{0} = g_{1}^{{ - l_{u} \cdot k_{u} + x^{\prime}}} \cdot g^{y^{\prime}}$,$v_{j} = g_{1}^{{z_{j} }} \cdot g^{{\varpi_{j} }} (1 \le j \le \ell )$,$y = g^{a}$, $u_{j} = g_{1}^{{\pi_{j} }} \cdot g^{{\omega_{j} }} (1 \le j \le c)$, $g_{1} = \zeta$, $\mu_{i} = g_{1}^{{x_{i} }} \cdot g^{{y_{i} }} (1 \le i \le l)$, $v_{0} = g_{1}^{{ - l_{w} \cdot k_{w} + z^{\prime}}} \cdot g^{\varpi ^{\prime}}$. The above public parameters are indistinguishable from the system global parameter $SysPara$.

**Queries:** Adversary $A$ can adaptively interact with the challenger $C$ and perform the following queries:

1. Extracting query: Adversary $A$ can adaptively submit its identity $ID_{i}$ to challenger $C$ to obtain its private key. Since challenger $C$ does not have the master key $msk$, challenger $C$ will respond to the query as follows: challenger $C$ computes $\widetilde{{u_{i} }}$ according to the Eq. (1) such that
  $$ W(\widetilde{{u_{i} }}) = x^{\prime} + \sum\limits_{j = 1}^{l} {x_{j} \cdot {\text{u}}_{i,j} - l_{u} \cdot k_{u} } ,T(\widetilde{{u_{i} }}) = y^{\prime} + \sum\limits_{j = 1}^{l} {y_{j} \cdot {\text{u}}_{i,j} } $$
  and hence,$$\mu_{0} \cdot \prod\nolimits_{j = 1}^{l} {\mu_{j}^{{{\text{u}}_{i,j} }} } = g_{1}^{{W(\widetilde{{u_{i} }})}} \cdot g^{{T(\widetilde{{u_{i} }})}}$$.
  If $W(\widetilde{{u_{i} }}) \ne 0$, challenger $C$ randomly chooses $\sigma_{i} \in {\mathbb{Z}}_{p}^{*}$ and computes the private key $sk_{i} = (sk_{i,1} ,sk_{i,2} )$, where
  $$ \left\{ \begin{aligned} & sk_{i,1} = y^{{ - \frac{{T(\widetilde{{u_{i} }})}}{{W(\widetilde{{u_{i} }})}}}} \cdot \left (\mu_{0} \cdot \prod\limits_{j = 1}^{l} {\mu_{j}^{{{\text{u}}_{i,j} }} } \right)^{{\sigma_{i} }} \hfill \\ & sk_{i,2} = y^{{ - \frac{1}{{W(\widetilde{{u_{i} }})}}}} \cdot g^{{\sigma_{i} }} \hfill \\ \end{aligned} \right. $$
  If lets the $\sigma_{i} ^{\prime} = \sigma_{i} - x/W(\widetilde{{u_{i} }})$, the private key $sk_{i}$ for the identity $ID_{i}$ can be proved to be legal and valid by verifying the following equation.
  $$ \begin{aligned} sk_{i,1} & = g_{1}^{x} \cdot (g_{1}^{{W(\widetilde{{u_{i} }})}} \cdot g^{{T(\widetilde{{u_{i} }})}} )^{{ - \frac{x}{{^{{W(\widetilde{{u_{i} }})}} }}}} \cdot (g_{1}^{{W(\widetilde{{u_{i} }})}} \cdot g^{{T(\widetilde{{u_{i} }})}} )^{{\sigma_{i} }} \hfill \\ & = g_{1}^{x} \cdot (g_{1}^{{W(\widetilde{{u_{i} }})}} \cdot g^{{T(\widetilde{{u_{i} }})}} )^{{\sigma_{i} - \frac{x}{{^{{W(\widetilde{{u_{i} }})}} }}}} \hfill \\ & = g_{1}^{x} \cdot \left (\mu_{0} \cdot \prod\limits_{j = 1}^{l} {\mu_{j}^{{{\text{u}}_{i,j} }} } \right)^{{\sigma_{i} ^{\prime}}} \hfill \\ \end{aligned} $$
  and
  $$ sk_{i,2} = g^{{\sigma_{i} - x/W(\widetilde{{u_{i} }})}} = g^{{\sigma_{i} ^{\prime}}} $$
  Therefore, the private key generated by the above method is indistinguishable from the real private key. If $W(\widetilde{{u_{i} }}) = 0$, the algorithm is terminated.
2. Delegation of authority query: Adversary $A$ may adaptively submit proof of authority $\Phi$ to the challenger $C$. Challenger $C$ can respond as follows.
  Challenger $C$ first calculates $\widetilde{{u_{o} }}$ according to Eq. (1) and second calculates $W(\widetilde{{u_{o} }})$. There are two scenarios for this condition:
  Case 1: $W(\widetilde{{u_{o} }}) \ne 0$. Challenger $C$ generates a private key for the DO according to the extraction query $sk_{o}$, generates a proof of delegation $\Gamma$ according to the proposed scheme and returns it to the adversary $A$.
  Case 2: $W(\widetilde{{u_{o} }}) = 0$. For a given proof of authorization $\Phi$, challenger $C$ computes $\widetilde{\varphi }$ according to Eq. (5) . Similarly, we make
    $$F(\widetilde{\varphi }) = z^{\prime} + \sum\limits_{j = 1}^{\ell } {z_{j} \cdot \phi_{j} - l_{w} \cdot k_{w} }, \quad J(\widetilde{\varphi }) = \varpi ^{\prime} + \sum\limits_{j = 1}^{\ell } {\varpi_{j} \cdot \phi_{j} }$$
    and so it follows that $v_{0} \cdot \prod\nolimits_{j = 1}^{\ell } {v_{j}^{{\phi_{j} }} } = g_{1}^{{F(\widetilde{\varphi })}} \cdot g^{{J(\widetilde{\varphi })}}$. If $F(\widetilde{\varphi }) \ne 0$, challenger $C$ randomly chooses $\sigma_{o} ,\sigma_{\phi } \in {\mathbb{Z}}_{p}^{*}$ and computes the proof of delegation $\Gamma ^{\prime} = (\alpha ^{\prime},\beta ^{\prime},\gamma ^{\prime})$, where
    $$\alpha ^{\prime} = sk_{o,1} \cdot y^{{ - \frac{{J(\widetilde{\varphi })}}{{F(\widetilde{\varphi })}}}} \cdot \left (v_{0} \cdot \prod\limits_{j = 1}^{\ell } {v_{j}^{{\phi_{j} }} } \right)^{{\sigma_{\phi } }}, \;\; \beta ^{\prime} = sk_{0,2}, \;\;  \gamma ^{\prime} = y^{{ - \frac{1}{{F(\widetilde{\varphi })}}}} \cdot g^{{\sigma_{\phi } }}$$
    If $\sigma_{\phi } ^{\prime} = \sigma_{\phi } - x/F(\widetilde{\varphi }),$, it follows from the following equation that the delegation proof
    $$ \Gamma ^{\prime} = (sk_{o,1} \cdot \left (v_{0} \cdot \prod\limits_{j = 1}^{\ell } {v_{j}^{{\phi_{j} }} } \right)^{{\sigma_{\phi } ^{\prime}}} ,sk_{o,2} ,g^{{\sigma_{\phi } ^{\prime}}} ) $$
    generated by the above method is indistinguishable from the real proof of delegation $\Gamma$. If $F(\widetilde{\varphi }) = 0$, then the algorithm is terminated.
3. File processing query: The challenger $C$ accepted the authorization proofs $(\Phi ,\Gamma )$ submitted by the adversary $A$.. The proposed scheme leads to a file processing query, which requires an extraction query and delegation of authority query. Challenger $C$ computes $W(\widetilde{{u_{o} }})$ and $F(\widetilde{\varphi })$, and if $W(\widetilde{{u_{o} }}) = 0$ and $F(\widetilde{\varphi }) = 0$, challenger $C$ terminates the algorithm. If $W(\widetilde{{u_{o} }}) \ne 0$ and $F(\widetilde{\varphi }) \ne 0$, then challenger $C$ executes the algorithm:
  Step A: Challenger $C$ generates a proof of delegation $\Gamma$ in the same way as in the delegation of authority query.
  Step B: First, challenger $C$ computes $\widetilde{{u_{p} }}$ according to Eq. (1) and determines whether $W(\widetilde{{u_{p} }})$ is zero, if $W(\widetilde{{u_{p} }}) = 0$, terminate the algorithm; otherwise, generate the private key $sk_{p}$ for PS as in the extraction query. Secondly, challenger $C$ randomly selects $\sigma_{F} \in {\mathbb{Z}}_{p}^{*}$ and computes $v_{F} = g^{{\sigma_{F} }}$, for each data block $\widetilde{m} = (m_{i,1} ,m_{i,2} , \ldots ,m_{i,c} )_{{\{ 1 \le i \le r\} }}$ randomly selects $\widehat{{\sigma_{i} }} \in {\mathbb{Z}}_{p}^{*}$ and sets
    $$ H_{3} (\Psi ) = \frac{{g^{{\widehat{{\sigma_{i} }}}} }}{{g_{1}^{{\sum\nolimits_{j = 1}^{c} {\pi_{j} \cdot m_{i,j} } }} \cdot g^{{\sum\nolimits_{j = 1}^{c} {\omega_{j} \cdot m_{i,j} } }} }} $$
    so we have $(H_{3} (\Psi ) \cdot \prod\nolimits_{j = 1}^{c} {u_{j}^{{m_{i,j} }} } )^{{\sigma_{F} }} = (g^{{\widehat{{\sigma_{i} }}}} )^{{^{{\sigma_{F} }} }}$.
    Finally, challenger $C$ computes the corresponding metadata tag $\vartheta_{i} = sk_{p,1} \cdot (g^{{\widehat{{\sigma_{i} }}}} )^{{\sigma_{F} }}$ for the data block $\widetilde{m}$. It follows that the metadata tags generated by the above approach are indistinguishable from the real generated metadata tags.
  Step C: After completing the above steps, challenger $C$ sends the processed file $M^{*} = \{ \{ \vartheta_{i} \}_{1 \le i \le c} ,\Gamma ,F_{id} \}$ to adversary $A$.

**Output:** Finally, if challenger $C$ does not terminate the algorithm, adversary $A$ outputs a processed file $\widehat{{M^{*} }}$ with non-negligible probability with respect to the proof of authorization $\widehat{\Phi }$. If the adversary $A$ wins the game, it implies that the proof of delegation $\widehat{\Gamma }$ is legally valid for the proof of authorization $\widehat{\Phi }$. The adversary $A$ has successfully compromised the proposed scheme with a negligible probability $\varepsilon$.

### **Theorem 4**

If the CDH difficulty assumption problem and PS's signature scheme $PS.Sign = (KeyGen,Sign,Verify)$ are computationally secure, then our scheme is secure and resilient against modification attacks. If the interrogated outsourced file contains data blocks that have been modified, then a CSP cannot respond as an audit by forging audit evidence.

### *Proof*

This proof process involves a series of safety games.

**Game 3** In this safety game, challenger $C$ and adversary $A$ will play as in **Game 1**.

**Game 4** This security game is similar to **Game 3** with the following differences. During the initialization phase of the protocol, the challenger $C$ has all the processed files. After completing the auditing protocol, the adversary $A$ outputs a proof, the proof of authorization $\widehat{\Phi }$, and the file $\widehat{{M^{*} }}$, which completes the validation of Eq. (10).

Suppose that adversary $A$ can win **Game 4** with non-negligible probability. Given a multiplicative cyclic group $G_{1}$ and a CDH difficulty assumption $(g,g^{\chi } ,\zeta )$, and the algorithm $\Lambda$ interacts with the adversary $A$ to compute $\zeta^{\chi }$. The process is as follows:

Algorithm $\Lambda$ is similar to the challenger $C$ in **Game 3** with the following differences:

1. First the algorithm $\Lambda$ randomly selects $x \in {\mathbb{Z}}_{p}^{*}$ and computes $y = g^{x}$,$g_{1} = \zeta$ and $msk = \zeta^{x}$. Then it randomly selects $\pi_{j} ,\omega_{j} \in {\mathbb{Z}}_{p}^{*} (1 \le j \le c)$ and computes $u_{j} = g_{1}^{{\pi_{j} }} \cdot g^{{\omega_{j} }}$.
2. The algorithm $\Lambda$ uses the function $H_{3} ( \cdot )$ to maintain all processed files and consistently answer all queries about them. Queries of the form $H_{3} (\Psi )$ will be responded to as follows.
3. Process a file *M* with an authorization certificate $\Phi$, compute $\widetilde{{u_{p} }} = H_{2} (ID_{p} )$ and extract the secret key of PS $sk_{p}$ according to the proposed scheme. Challenger $C$ randomly selects $\rho \in {\mathbb{Z}}_{p}^{*}$ for the file *M* and computes $v_{F} = (g^{\alpha } )^{\rho }$, which is compared with the proposed scheme to obtain $\sigma_{F} = \alpha \cdot \rho$. For each data block $\widetilde{m} = (m_{i,1} ,m_{i,2} , \ldots ,m_{i,c} )_{{\{ 1 \le i \le r\} }}$ randomly selects $\widehat{{\sigma_{i} }} \in {\mathbb{Z}}_{p}^{*}$ and sets
  $$ H_{3} (\Psi ) = \frac{{g^{{\widehat{{\sigma_{i} }}}} }}{{g_{1}^{{\sum\nolimits_{j = 1}^{c} {\pi_{j} \cdot m_{i,j} ^{\prime}} }} \cdot g^{{\sum\nolimits_{j = 1}^{c} {\omega_{j} \cdot m_{i,j} ^{\prime}} }} }} $$
  so we have $(H_{3} (\Psi ) \cdot \prod\nolimits_{j = 1}^{c} {u_{j}^{{m_{i,j} ^{\prime}}} } )^{{\sigma_{F} }} = (g^{{\widehat{{\sigma_{i} }}}} )^{{^{{\sigma_{F} }} }} = (v_{F} )^{{\widehat{{\sigma_{i} }}}}$.
  Finally, challenger $C$ computes the corresponding metadata tag $\vartheta_{i} = sk_{p,1} \cdot (v_{F} )^{{\widehat{{\sigma_{i} }}}}$ for the data block $\widetilde{m}$. It follows that the metadata tags generated by the above approach are indistinguishable from the real generated metadata tags.
4. The algorithm $\Lambda$ interacts with adversary $A$ to execute an integrity auditing protocol. According to **Game 4**, if the metadata aggregation tag $\widehat{\vartheta }$ output by adversary $A$ during the auditing process is not equal to the desired metadata aggregation tag $\vartheta$, the auditing protocol is terminated.

Let $\{ \vartheta_{i} \}_{1 \le i \le c}$ be the set of aggregated tags of the data blocks questioned in the audit evidence $\widehat{proof}$, so that we have.

11 $$ \begin{aligned} e(\widehat{\vartheta },g) & = e((g_{1}^{x} )^{{\sum\limits_{{i \in S_{F} }} {s_{i} } }} \cdot \left( \left(\mu_{0} \cdot \prod\limits_{j = 1}^{l} {\mu_{j}^{{{\text{u}}_{p,j} }} } \right)^{{\sigma_{p} }} \right)^{{\sum\limits_{{i \in S_{F} }} {s_{i} } }} \hfill \\ & \quad \cdot  \left(\prod\limits_{{i \in S_{f} }} { \left(H_{3}  \left(\widehat{\Psi } \right)^{{s_{i} }} \cdot \prod\limits_{j = 1}^{c} {u_{j}^{{\sum\limits_{{i \in S_{F} }} {s_{i} \cdot m_{i,j} ^{\prime}} }} }  \right)^{{\sigma_{F} }} } ,g \right) \hfill \\ &=  \left(e \left(y,g_{1}  \right) \cdot e \left(sk_{p,1}^{{\frac{1}{{\sigma_{p} }}}} ,sk_{p,2}  \right) \right)^{{\sum\limits_{{i \in S_{F} }} {s_{i} } }} \cdot e \left(\prod\limits_{{i \in S_{F} }} {H_{3}  \left(\widehat{\Psi } \right)^{{s_{i} }} \cdot \prod\limits_{j = 1}^{c} {u_{j}^{{\widehat{{\eta_{j} }}}} } } ,v_{F}  \right) \hfill \\ \end{aligned} $$

Based on the correct audit proof *proof*, we have

12 $$ \begin{aligned} e (\widehat{\vartheta },g ) & = e ( (g_{1}^{x}  )^{{\sum\limits_{{i \in S_{F} }} {s_{i} } }} \cdot  \left( \left(\mu_{0} \cdot \prod\limits_{j = 1}^{l} {\mu_{j}^{{{\text{u}}_{p,j} }} }  \right)^{{\sigma_{p} }}  \right)^{{\sum\limits_{{i \in S_{F} }} {s_{i} } }} \hfill \\ & \quad  \cdot  \left(\prod\limits_{{i \in S_{f} }} { \left(H_{3}  (\widehat{\Psi } )^{{s_{i} }} \cdot \prod\limits_{j = 1}^{c} {u_{j}^{{\sum\limits_{{i \in S_{F} }} {s_{i} \cdot m_{i,j} ^{\prime}} }} } \right )^{{\sigma_{F} }} } ,g \right) \hfill \\ & =  (e (y,g_{1}  ) \cdot e (sk_{p,1}^{{\frac{1}{{\sigma_{p} }}}} ,sk_{p,2}  ) )^{{\sum\limits_{{i \in S_{F} }} {s_{i} } }} \cdot e \left (\prod\limits_{{i \in S_{F} }} {H_{3}  (\widehat{\Psi } )^{{s_{i} }} \cdot \prod\limits_{j = 1}^{c} {u_{j}^{{\eta_{j} }} } } ,v_{F}  \right) \hfill \\ \end{aligned} $$

Comparison of the above equations shows that for every $j \in [1,c]$,$\widehat{{\eta_{j} }} = \eta_{j}$ cannot hold, otherwise the generated metadata aggregation tag $\widehat{\vartheta } = \vartheta$. Define on the set $S_{F}$ that there exists at least one $\Delta \eta_{j} = \widehat{{\eta_{j} }} - \eta_{j}$ that is not zero. We assume $v_{F} = (g^{\chi } )^{{\rho_{F} }}$ and divide the above two equations to get

$$ e({{\widehat{\vartheta }}/{\vartheta ,g}}) = e \left(\prod\limits_{j = 1}^{c} {u_{j}^{{\Delta \eta_{j} }} } ,v_{F} \right) = e \left(\prod\limits_{j = 1}^{c} {(g_{1}^{{\pi_{j} }} \cdot g^{{\omega_{j} }} )^{{\Delta \eta_{j} }} } ,(g^{\chi } )^{{\rho_{F} }} \right) $$

which simplifies to

$$ e(\widehat{\vartheta } \cdot \vartheta^{ - 1} \cdot (g^{\chi } )^{{ - \rho_{F} \cdot \sum\limits_{j = 1}^{c} {\omega_{j} \cdot \Delta \eta_{j} } }} ,g) = e(\zeta ,g^{\chi } )^{{\rho_{F} \cdot \sum\limits_{j = 1}^{c} {\pi_{j} \cdot \Delta \eta_{j} } }} $$

Thus we get the method for solving the CDH difficulty assumption as follows:

$$ \zeta^{\chi } = (\widehat{\vartheta } \cdot \vartheta \cdot (g^{\chi } )^{{ - \rho_{F} \cdot \sum\limits_{j = 1}^{c} {\omega_{j} \cdot \Delta \eta_{j} } }} )^{{\frac{1}{{\rho_{F} \cdot \sum\limits_{j = 1}^{c} {\pi_{j} \cdot \Delta \eta_{j} } }}}} $$

The probability that $\rho_{F} \cdot \sum\nolimits_{j = 1}^{c} {\pi_{j} \cdot \Delta \eta_{j} } \ne 0$. Since there exists at least one $\Delta \eta_{j} = \widehat{{\eta_{j} }} - \eta_{j}$ that is not zero, both $\rho_{F}$ and $\pi_{j} (1 \le j \le c)$ are random values and with probability $\Pr [\rho_{F} \cdot \sum\nolimits_{j = 1}^{c} {\pi_{j} \cdot \Delta \eta_{j} = 0} ] = {1/p}$.

If the difference between the probabilities of adversary $A$ winning the game on **Game 3** and **Game 4** is non-negligible, then the above algorithm $\Lambda$ can be constructed to solve the CDH problem.

**Game 5** This secure game is similar to **Game 4** with the following differences. During the initialization phase of the protocol, the challenger $C$ has all the processed documents. After completing the auditing protocol, the adversary $A$ outputs a proof, the proof of authorization $\widehat{\Phi }$, and the file $\widehat{{M^{*} }}$, which is capable of completing the validation of Eq. (10).

Suppose adversary $A$ wins **Game 5** with non-negligible probability. Given a DL difficulty assumption $(g,\zeta )$, the algorithm $\Lambda$ interacts with adversary $A$ to compute $\chi$ such that it satisfies $\zeta = g^{\chi }$.

The algorithm $\Lambda$ is similar to challenger $C$ in **Game 4** with the following differences:

1. Process a file *M* with an authorization proof $\Phi$, calculate $\widetilde{{u_{p} }} = H_{2} (ID_{p} )$ and extract the secret key $sk_{p}$ of PS. According to the proposed scheme. Process the file *M* using the parameter $u_{j} = g_{1}^{{\pi_{j} }} \cdot g^{{\omega_{j} }} (1 \le j \le c)$, where $\pi_{j} ,\omega_{j} \in {\mathbb{Z}}_{p}^{*}$.
2. The algorithm $\Lambda$ interacts with adversary $A$ to execute the integrity auditing protocol. According to **Game 5**, adversary $A$ terminates the auditing protocol if the data tag $\{ \widehat{{\eta_{j} }}\}_{1 \le j \le c}$ output by the adversary during the auditing process is not equal to the desired data tag $\{ \eta_{j} \}_{1 \le j \le c}$.

According to **Game 4** we have $\widehat{\vartheta } = \vartheta$. From Eqs. (11) and (12) we have that

$$ e \left(\prod\limits_{{i \in S_{F} }} {H_{3} \left(\widehat{\Psi }\right)^{{s_{i} }} \cdot \prod\limits_{j = 1}^{c} {u_{j}^{{\widehat{{\eta_{j} }}}} } } ,v_{F} \right) = e\left(\prod\limits_{{i \in S_{F} }} {H_{3} \left(\widehat{\Psi }\right)^{{s_{i} }} \cdot \prod\limits_{j = 1}^{c} {u_{j}^{{\eta_{j} }} } } ,v_{F} \right) $$

a further derivation yields $\prod\nolimits_{j = 1}^{c} {u_{j}^{{\widehat{{\eta_{j} }}}} } = \prod\nolimits_{j = 1}^{c} {u_{j}^{{\eta_{j} }} }$, defined on the set $S_{F}$, and there exists at least one $\Delta \eta_{j} = \widehat{{\eta_{j} }} - \eta_{j}$ that is not zero. We have

$$ \prod\limits_{j = 1}^{c} {u_{j}^{{\Delta \eta_{j} }} } = \prod\limits_{j = 1}^{c} {(g_{1}^{{\pi_{j} }} \cdot g^{{\omega_{j} }} )^{{\Delta \eta_{j} }} } = \zeta^{{\sum\limits_{j = 1}^{c} {\pi_{j} \cdot \Delta \pi_{j} } }} \cdot g^{{\sum\limits_{j = 1}^{c} {\omega_{j} \cdot \Delta \pi_{j} } }} = 1 $$

so we get the solution to the DL difficulty assumption method as follows:

$$ \chi = - \left( {\sum\limits_{j = 1}^{c} {\omega_{j} \cdot \Delta \eta_{j} } } \right) \cdot \left( {\sum\limits_{j = 1}^{c} {\pi_{j} \cdot \Delta \eta_{j} } } \right) $$

where $$\sum\nolimits_{j = 1}^{c} {\pi_{j} \cdot \Delta \eta_{j} } \ne 0$$.

Since there exists at least one $\Delta \eta_{j} = \widehat{{\eta_{j} }} - \eta_{j}$ that is not zero, $\pi_{j} (1 \le j \le c)$ is a random value and with probability $\Pr [\sum\nolimits_{j = 1}^{c} {\pi_{j} \cdot \Delta \eta_{j} } \ne 0] = {1/p}$.

If the difference in probabilities between the adversary $A$ winning in the **Game 4** and **Game 5** is non-negligible, then the above algorithm $\Lambda$ can be constructed to address the difficulty of the DL problem.

## Performance analysis

In this section, we evaluate the performance of the proposed scheme. Firstly, we analyze the computation and communication overhead from the theoretical level. To simplify the presentation,$T_{P}$ denote the bilinear mapping operation,$T_{Mul}$,$T_{Add}$,$T_{Exp}$, and $T_{H}$ denote the multiplication, addition, exponentiation, hash mapping operations on $G_{1}$, respectively.

### Theoretical analysis

During the data block signing phase, the computational overhead for PS to compute the data tag is $n(T_{H} + 2T_{Mul} + T_{Exp} )$. In the audit evidence generation phase, the CSP computes the audit proof with a total computational overhead of $n(T_{Add} + 2T_{Mul} + T_{Exp} )$. In the proof verification phase, TPA validates the audit evidence with a computational overhead of $4T_{P} + c(T_{H} + 2T_{Exp} + T_{Mul} )$. The results of the comparative analysis with other schemes are shown in Table 2.

**Table 2:** Comparison of computational overhead.

Schemes	TagGen	ProofGen	ProofVerify
Jalil et al.^17^	$n(T_{H} + 2T_{Mul} + 2T_{Exp} )$	$2n(T_{H} + T_{Mul} + 2T_{Exp} )$	$2T_{P} + c(T_{H} + 2T_{Exp} + 2T_{Mul} )$
Guo et al.^19^	$n(T_{H} + 2T_{Mul} + 3T_{Exp} )$	$n(T_{Add} + 2T_{Exp} + 2T_{Mul} )$	$2T_{P} + c(2T_{Exp} + 2T_{Mul} )$
Rao et al.^21^	$n(T_{H} + T_{Mul} + 3T_{Exp} )$	$(n + 1)T_{Add} + n(2T_{Mul} + 3T_{Exp} )$	$c(4T_{Mul} + 3T_{Exp} )$
Ours scheme	$n(T_{H} + 2T_{Mul} + T_{Exp} )$	$n(T_{Add} + 2T_{Mul} + T_{Exp} )$	$4T_{P} + c(T_{H} + 2T_{Exp} + T_{Mul} )$

In our proposed scheme, we focus only on the communication overhead imposed by the audit challenge and proof generation phases. In order to ensure a 160-bit level of security for the system, a 512-bit $G_{1}$ parameter size and a 160-bit ${\mathbb{Z}}_{p}^{*}$ parameter setting are chosen in our scheme, respectively. In the challenge generation phase of our scheme, the audit challenge $chal = (i,s_{i} )_{{i \in S_{F} }}$ is initiated by the TPA to the CSP, and its communication cost is $2|p|$, and the communication cost incurred by the CSP returning proof $proof = (\vartheta ,\{ \eta_{j} \}_{1 \le j \le c} )$ to the TPA is $|p| + |q|$. In addition, the data structure of this paper's scheme is stored on the TPA side, which requires lower communication costs than storing it on the CSP side. In Table 3, we compared the communication overhead incurred by the proposed scheme with other cloud data auditing schemes when sending audit challenges in the challenge generation phase and audit proofs in the proof generation phase.

**Table 3:** Comparison of communication overhead.

Schemes	ChalGen	ProofGen
Jalil et al.^17^	$c(|p| + |q|)$	$2|p|$
Guo et al.^19^	$c(2|p| + |q|)$	$|p| + 2|q|$
Rao et al.^21^	$c(2|p| + |q|)$	$3|p| + |q|$
Ours scheme	$2|p|$	$|p| + |q|$

### Experimental analysis

The experimental environment is configured as an AMD Ryzen7 5800H with Radeon Graphics 3.2 GHz RAM32GHz laptop, and all the simulations are implemented on the Ubuntu system. Using the Pairing Based Cryptography PBC and the GUN Multiple arithmetic Precision to implement the corresponding cryptographic operations. Python was used for data processing and experimental result analysis. In our experiments, we chose 2000 data blocks, each with a size of 8 KB, and the length of *p* was chosen to be 160 bits.

#### Time overhead in the key generation and proof of authorization generation phase

**Figure 5:**
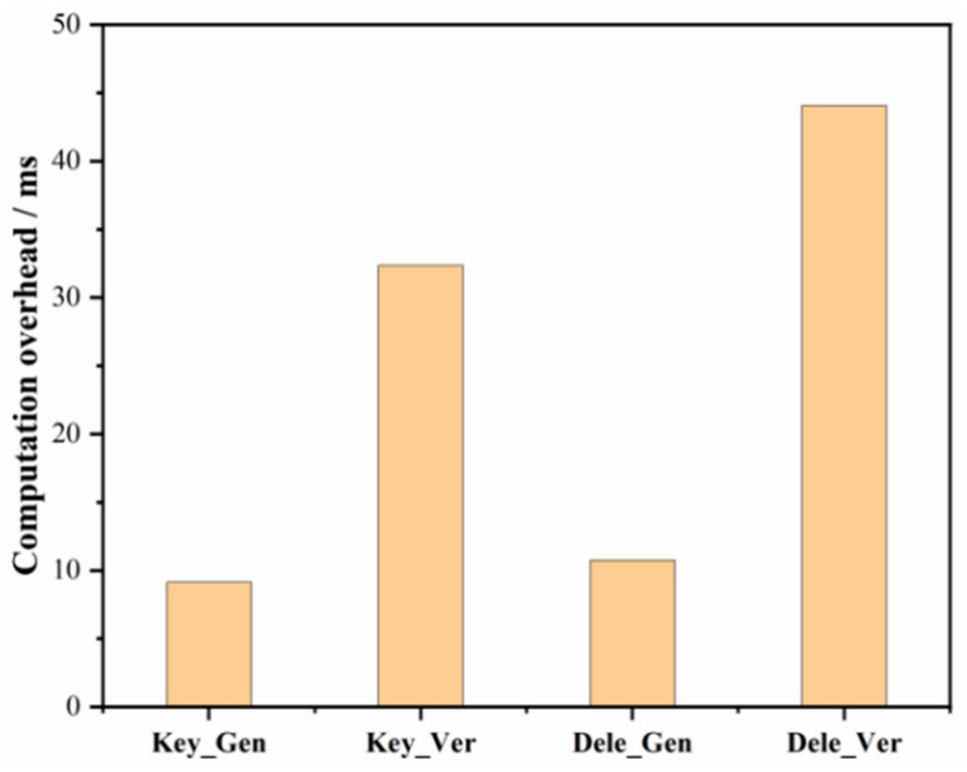
Key generation verification and proof of authority generation verification time overheads.

The time overhead performance of generating and verifying the private key for a particular user and the time overhead performance of generating and verifying the proof of delegation are shown in Fig. 5. The time consumed for key generation and verification, and attorney certificate generation and verification is about 9.13 ms, 32.34 ms, 10.75 ms, and 44.07 ms, respectively, which is negligible for deployment in real applications.

#### The computation overhead at the TPA and CSP sides

**Figure 6:**
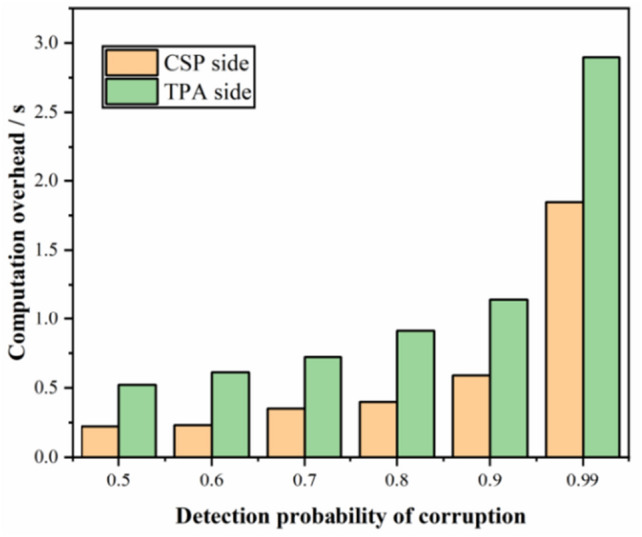
The computation overhead at the TPA and CSP sides under different detection probabilities of corruption.

**Figure 7:**
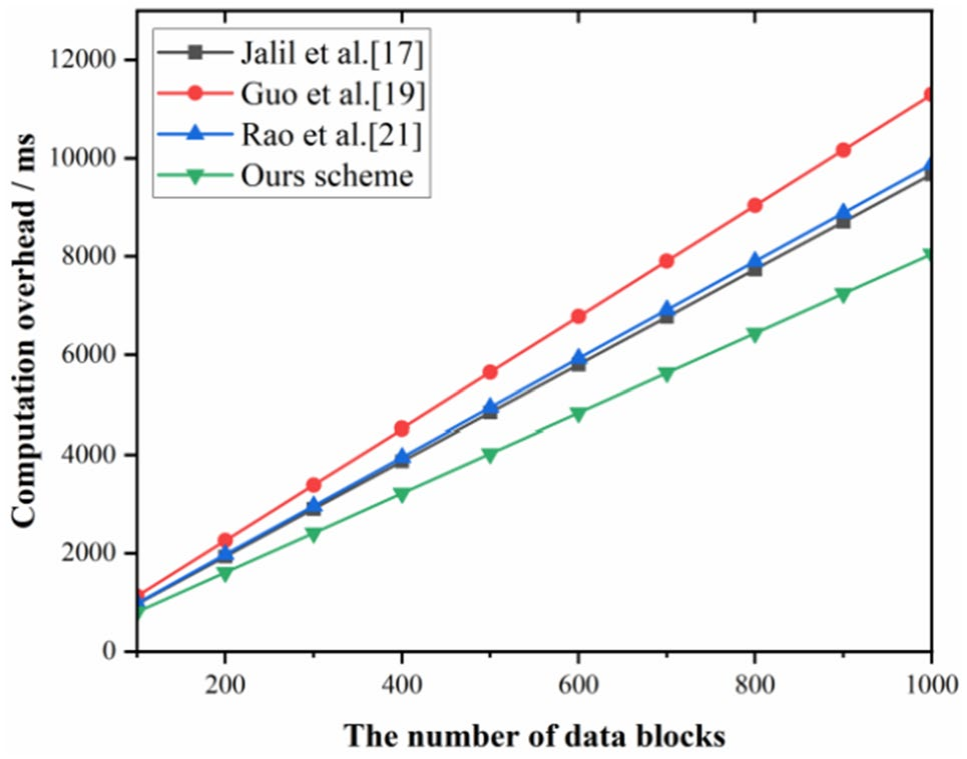
Data signature time overhead.

Figure 6 shows the time overhead required to audit an outsourced file with a corruption rate of 1%; we simulate the time overhead required to audit an outsourced file under different corruption detection probabilities, i.e., 0.5, 0.6, 0.7, 0.8, 0.9, and 0.99. The simulation results in Fig. 7 show that our scheme incurs time overhead at both the TPA and CSP sides when executing the auditing protocol. For achieving a detection probability of 0.99, the TPA in the scheme can do it in less than 3 s. In the scheme, the computation overhead at the TPA side is higher than at the CSP side, which is caused by the higher number of bilinear mapping operations employed at the TPA side.

#### Time overhead in the data signature generation phase

Figure 7 illustrates the time overhead performance curve of the proposed scheme with scheme^17^, scheme^19^, and scheme^21^ in the data block signature generation phase. The proposed scheme has fewer exponential operations compared to the scheme^17^and scheme^19^, so its computational overhead is lower. Whereas scheme^19^ has more multiplicative and exponential operations, and its computational overhead is higher.

#### Time overhead in the data proof generation phase

**Figure 8:**
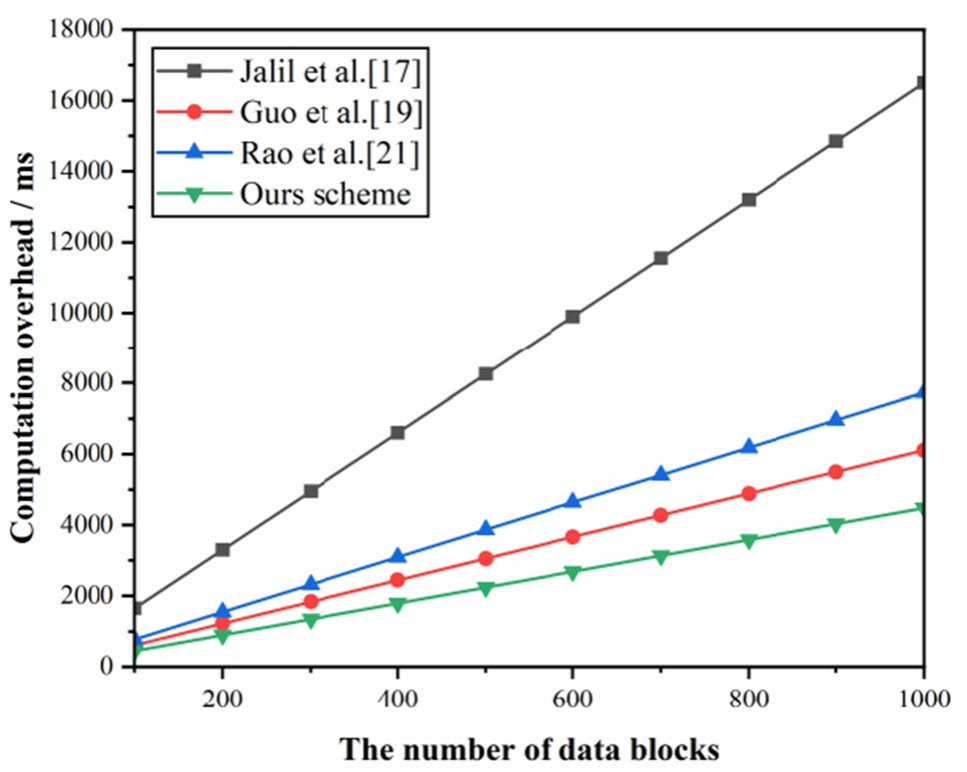
Proof generation time overhead.

The CSP generates relevant data proof time performance curves based on challenge audits, as shown in Fig. 8. From the figure, it can be observed that the proof generation time for each scheme increases linearly as the number of queried data blocks increases. Comprehensively checking all data blocks in the cloud increases the computational burden. Therefore, to improve efficiency, we propose to specify 460 data blocks in the query audit message, which is sufficient to achieve 99% probability of data corruption or tampering for a real cloud data auditing system. In this case, the computational overhead of the proposed scheme is only about 1.84 s.

#### Time overhead of the data proof verification phase

**Figure 9:**
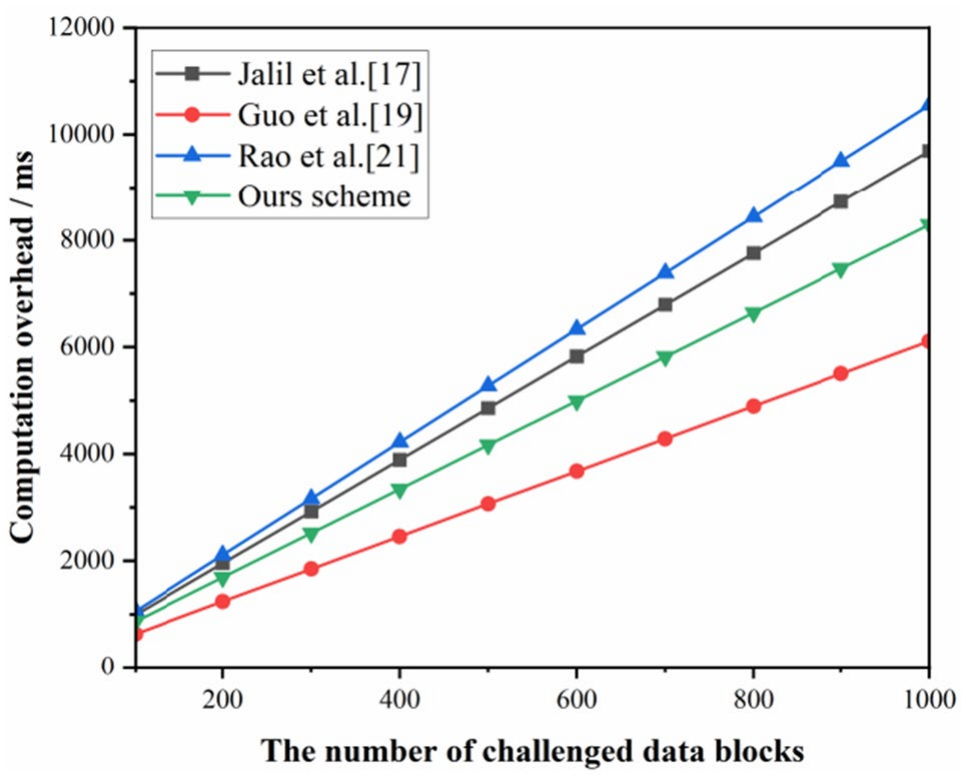
Proof verification time overhead.

The performance curves of the time overhead generated by TPA during the data evidence validation phase are shown in Fig. 9. As shown in Fig. 9, all the validation data computation overheads are linear and increase with the number of challenge data blocks. However, compared to schemes^17^ and^21^, our scheme has fewer multiplicative and exponential operations and uses less validation time. Compared with the scheme^19^, our scheme uses more pairwise operations in the verification phase and is, therefore, slightly more efficient than the scheme^19^. The time overhead is about 8.29 s in verifying 1000 data blocks.

#### Time overhead of the dynamic update phase

**Figure 10:**
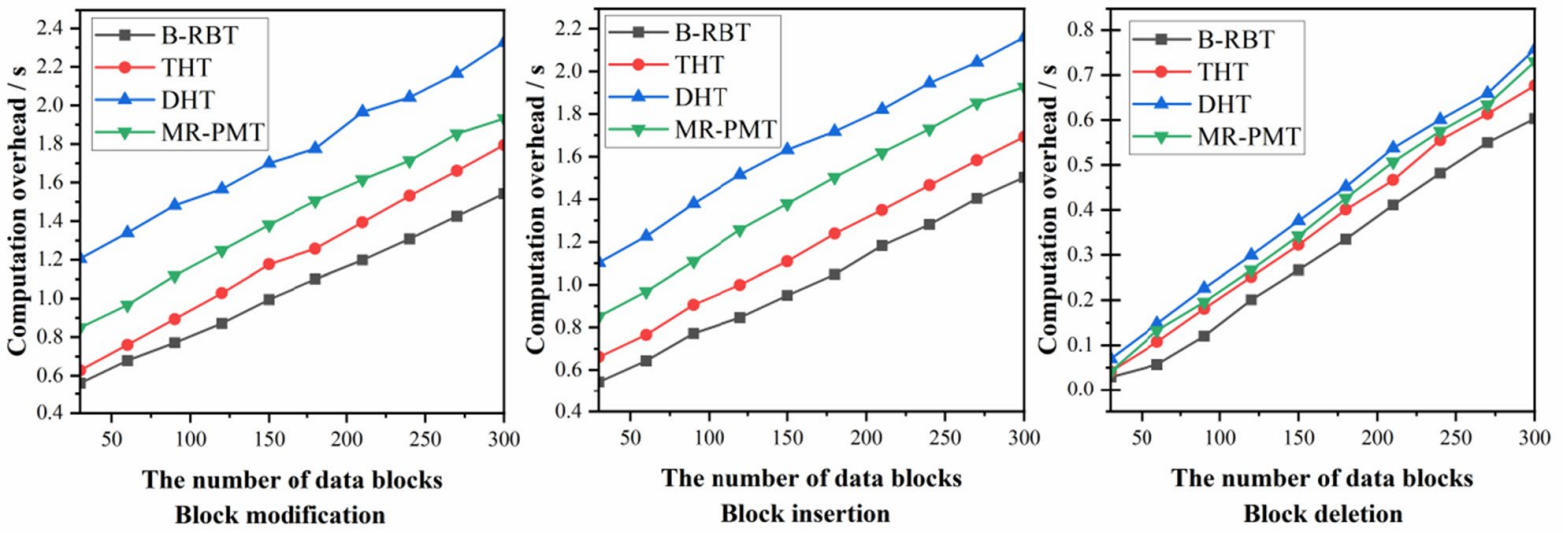
Data update time overhead.

The B-RBT data structure of this scheme is compared with THT Scheme^23^, DHT Scheme^26^, and MR-PMT Scheme^27^, and the time cost of our scheme is lower. Where the time complexity of insertion and modification of data blocks for both the B-RBT structure and THT structure is $O(1)$, but the time complexity of lookup for the THT structure is $O(\log n)$. Secondly, the structure time complexity of DHT and MR-PMT are both $O(\log n)$, which takes more time overhead, the time overhead required for data update operation of each scheme is shown in Fig. 10.

## Conclusion

In this paper, we propose a secure identity-based controlled delegation of outsourced data integrity auditing scheme. The data owner generates a proof of authorization delegation and appoints an proxy to help him/her upload files to the cloud. The processing and outsourcing of selected files on behalf of the data owner may only be carried out by an authorized proxy server. The identity-based controlled delegation and public auditing features make our scheme superior to existing cloud data auditing schemes. In order to satisfy the data update efficiency, the B-RBT data structure is introduced to complete the data update in a constant time. The scheme has been deemed secure and effective based on security analysis and experimental outcomes.

## Data Availability

Data sharing not applicable to this paper as no datasets were generated or analyzed during the current study.

## References

[1] Vineela, A., Kasiviswanath, N. & Bindu, C. S. Data integrity auditing scheme for preserving security in cloud based big data. In *2022 6th International Conference on Intelligent Computing and Control Systems (ICICCS)*, 609–613 (IEEE, 2022).

[2] Shen, J., Liu, D., He, D., Huang, X. & Xiang, Y. Algebraic signatures-based data integrity auditing for efficient data dynamics in cloud computing. *IEEE Trans. Sustain. Comput.* **5**(2), 161–173 (2020). 10.1109/TSUSC.2017.2781232

[3] Yan, H. & Gui, W. Efficient identity-based public integrity auditing of shared data in cloud storage with user privacy preserving. *IEEE Access* **9**, 45822–45831 (2021). 10.1109/ACCESS.2021.3066497

[4] Yang, Y., Chen, Y., Chen, F. & Chen, J. Identity-based cloud storage auditing for data sharing with access control of sensitive information. *IEEE Internet Things J.* **9**(13), 10434–10445 (2022). 10.1109/JIOT.2021.3121678

[5] Sengupta, B., Dixit, A. & Ruj, S. Secure cloud storage with data dynamics using secure network coding techniques. *IEEE Trans. Cloud Comput.* **10**(3), 2090–2101 (2022). 10.1109/TCC.2020.3000342

[6] Li, Y. *et al.* Fuzzy identity-based data integrity auditing for reliable cloud storage systems. *IEEE Trans. Depend. Secure Comput.* **16**(1), 72–83 (2019). 10.1109/TDSC.2017.2662216

[7] Li, J., Yan, H. & Zhang, Y. Efficient identity-based provable multi-copy data possession in multi-cloud storage. *IEEE Trans. Cloud Comput.* **10**(1), 356–365 (2022). 10.1109/TCC.2019.2929045

[8] Yang, K., Jia, X. & Ren, K. Secure and verifiable policy update outsourcing for big data access control in the cloud. *IEEE Trans. Parallel Distrib. Syst.* **26**(12), 3461–3470 (2015). 10.1109/TPDS.2014.2380373

[9] Li, J., Yan, H. & Zhang, Y. Identity-based privacy preserving remote data integrity checking for cloud storage. *IEEE Syst. J.* **15**(1), 577–585 (2021). 10.1109/JSYST.2020.2978146

[10] Bian, G., Zhang, R. & Shao, B. Identity-based privacy preserving remote data integrity checking with a designated verifier. *IEEE Access* **10**, 40556–40570 (2022). 10.1109/ACCESS.2022.3166920

[11] Ateniese, G. *et al.* Provable data possession at untrusted stores. In *Proceedings of the 14th ACM conference on Computer and Communications Security*, 598–609 (ACM, 2007).

[12] Yang, K. & Jia, X. Data storage auditing service in cloud computing: Challenges, methods and opportunities. *World Wide Web* **15**(4), 409–428 (2012). 10.1007/s11280-011-0138-0

[13] Yu, Y. *et al.* Identity-based remote data integrity checking with perfect data privacy preserving for cloud storage. *IEEE Trans. Inform. Forensic Secur.* **12**(4), 767–778 (2017). 10.1109/TIFS.2016.2615853

[14] Zheng, X. & Cai, Z. Privacy-preserved data sharing towards multiple parties in industrial IoTs. *IEEE J. Select. Areas Commun.* **38**(5), 968–979 (2020). 10.1109/JSAC.2020.2980802

[15] Ping, Y., Zhan, Y., Lu, K. & Wang, B. Public data integrity verification scheme for secure cloud storage. *Information* **11**(9), 409 (2020). 10.3390/info11090409

[16] Shen, J., Shen, J., Chen, X., Huang, X. & Susilo, W. An efficient public auditing protocol with novel dynamic structure for cloud data. *IEEE Trans. Inform. Forensic Secur.* **12**(10), 2402–2415 (2017). 10.1109/TIFS.2017.2705620

[17] Jalil, B. A., Hasan, T. M., Mahmood, G. S. & Noman Abed, H. A secure and efficient public auditing system of cloud storage based on BLS signature and automatic blocker protocol. *J. King Saud Univ. Comput. Inf. Sci.* **34**(7), 4008–4021 (2022).

[18] Ji, Y., Shao, B., Chang, J., Xu, M. & Xue, R. Identity-based remote data checking with a designated verifier. *J. Cloud Comp.* **11**(1), 7 (2022). 10.1186/s13677-022-00279-5

[19] Guo, W. *et al.* Outsourced dynamic provable data possession with batch update for secure cloud storage. *Future Gener. Comput. Syst.* **95**, 309–322 (2019). 10.1016/j.future.2019.01.009

[20] Yang, A., Xu, J., Weng, J., Zhou, J. & Wong, D. S. Lightweight and privacy-preserving delegatable proofs of storage with data dynamics in cloud storage. *IEEE Trans. Cloud Comput.* **9**(1), 212–225 (2021). 10.1109/TCC.2018.2851256

[21] Rao, L., Zhang, H. & Tu, T. Dynamic outsourced auditing services for cloud storage based on batch-leaves-authenticated Merkle hash tree. *IEEE Trans. Serv. Comput.* **13**(3), 451–463 (2020). 10.1109/TSC.2017.2708116

[22] Zhang, X., Zhao, J., Xu, C., Wang, H. & Zhang, Y. DOPIV: Post-quantum secure identity-based data outsourcing with public integrity verification in cloud storage. *IEEE Trans. Serv. Comput.* **15**(1), 334–345 (2022). 10.1109/TSC.2019.2942297

[23] Thangavel, M. & Varalakshmi, P. Enabling ternary hash tree based integrity verification for secure cloud data storage. *IEEE Trans. Knowl. Data Eng.* **32**(12), 2351–2362 (2020). 10.1109/TKDE.2019.2922357

[24] Zou, J., Sun, Y. & Li, S. Dynamic provable data possession based on ranked Merkle hash tree. In *2016 International Conference on Identification, Information and Knowledge in the Internet of Things*, 4–9 (IIKI), (IEEE, 2016).

[25] Hariharasitaraman, S. & Balakannan, S. P. A dynamic data security mechanism based on position aware Merkle tree for health rehabilitation services over cloud. *J. Ambient Intell. Hum. Comput.*10.1007/s12652-019-01412-0 (2019). 10.1007/s12652-019-01412-0

[26] Li, R. *et al.* Efficient certificateless public integrity auditing of cloud data with designated verifier for batch audit. *J. King Saud Univ. Comput. Inf. Sci.* **34**(10), 8079–8089 (2022).

[27] Peng, S., Zhou, F., Li, J., Wang, Q. & Xu, Z. Efficient, dynamic and identity-based Remote Data Integrity Checking for multiple replicas. *J. Netw. Comput. Appl.* **134**, 72–88 (2019). 10.1016/j.jnca.2019.02.014

[28] Kim, I., Susilo, W., Baek, J. & Kim, J. Harnessing policy authenticity for hidden ciphertext policy attribute-based encryption. *IEEE Trans. Depend. Secure Comput.* **19**(3), 1856–1870 (2022). 10.1109/TDSC.2020.3040712

[29] Shen, W. *et al.* Data integrity auditing without private key storage for secure cloud storage. *IEEE Trans. Cloud Comput.* **9**(4), 1408–1421 (2021). 10.1109/TCC.2019.2921553

[30] Liu, Z., Liu, Y., Yang, X. & Li, X. Integrity auditing for multi-copy in cloud storage based on red-black tree. *IEEE Access* **9**, 75117–75131 (2021). 10.1109/ACCESS.2021.3079143

[31] Elmasry, A., Kahla, M., Ahdy, F. & Hashem, M. Red-black trees with constant update time. *Acta Inform.* **56**(5), 391–404 (2019). 10.1007/s00236-019-00335-9

[32] Paterson, K. G. & Schuldt, J. C. N. Efficient identity-based signatures secure in the standard model. In *Information Security and Privacy*, (eds Batten, L. M. & Safavi-Naini, R.) Lecture Notes in Computer Science, vol. 4058, 207–222 (Springer, 2006).

